# Greenland and Svalbard glaciers host unknown basidiomycetes: the yeast *Camptobasidium arcticum* sp. nov. and the dimorphic *Psychromyces glacialis* gen. and sp. nov.

**DOI:** 10.1099/ijsem.0.004655

**Published:** 2021-01-27

**Authors:** Laura Perini, Kristina Andrejašič, Cene Gostinčar, Nina Gunde-Cimerman, Polona Zalar

**Affiliations:** ^1^​ Department of Biology, Biotechnical Faculty, University of Ljubljana, Jamnikarjeva 101, SI-1000 Ljubljana, Slovenia; ^2^​ Lars Bolund Institute of Regenerative Medicine, BGI-Qingdao, Qingdao 266555, PR China

**Keywords:** psychrophiles, yeast, filamentous, Svalbard, Greenland Ice Sheet, glacial ice, Microbotryomycetes

## Abstract

Sampling campaigns in Greenland and Svalbard were executed to explore fungal diversity in cold habitats. Three very abundant groups of strains were discovered, consisting either of recently described or of yet-undescribed psychrophilic and oligotrophic yeasts and dimorphic fungi, accounting for around 50 % of the total cultivable diversity of basidiomycetes in our studies. The occurrence of these taxa has also been demonstrated by culture-independent methods. Based on phylogenetic analyses of ribosomal gene cluster sequences (D1/D2 domains of 28S (LSU), 18S (SSU), ITS with 5.8S rDNA) and sequences of protein-coding genes for elongation factor one alpha (*TEF*), cytochrome b (*CYTB*) and two subunits of the RNA polymerase II (*RPB1* and *RPB2*) obtained from pure cultures, the isolated taxa presented in this study belong to Basidiomycota, subphylum Pucciniomycotina, class Microbotryomycetes, family Camptobasidiaceae. The dataset of the sequences supported the recognition of three species: *Camptobasidium gelus*, *Camptobasidium arcticum* sp. nov. (ex-type strain EXF-12713) and *Psychromyces glacialis* gen. and sp. nov. (ex-type strain EXF-13111). *Camptobasidium gelus* was found in the Svalbard and Greenland samples, while representatives of the here proposed new species, *C. arcticum*, were found only in the Greenland Ice Sheet. *Psychromyces* gen. nov. was erected for the dimorphic/filamentous isolates found in Svalbard and Greenland glacial environments. The taxon, for which the invalid name ‘*Rhodotorula svalbardensis*’ has been used, belongs to this genus. Based on ribosomal genes, *Camptobasidium arcticum* and *Psychromyces glacialis* are related, phylogenetically most closely related to the genera *Glaciozyma* and *Cryolevonia*. Seven genes phylogeny restricted to taxa with available sequences, supported the placement of *Psychromyces* to Camptobasidiaceae.

Basidiomycetous yeasts have a widespread distribution in cold environments, particularly in polar areas [1]. Psychrotolerant and psychrophilic species can be endemic or cosmopolitan [2]. Due to their ability to survive in extreme conditions they have become model organisms in searches for potentially novel secondary metabolites and in studies addressing the evolution of life strategies at low temperatures [3–8]. In recent years, the diversity of yeast species in glacial habitats of polar (Antarctic and Arctic) and non-polar (e.g. Alpine) areas has been investigated with culture-dependent and independent methods [9–18]. These studies reported considerable numbers of new taxa, for instance, in *Bannozyma—B. arctica* [19]; *Mrakia – M. robertii*, *M. blollopsis*, *M. psychrophila* [20, 21]; *Rhodotorula—R. arctica* [22]; *Phenoliferia – P. psychrophenolica*, *P. psychrophila, P. glacialis* [23]; *Glaciozyma—G. watsonii*, *G.martinii*, *G. antarctica*, *G. litorale* [24, 25]; *Camptobasidium – C. gelus* [18]; and *Cryolevonia—C. schafbergensis*, *C. giraudoae* [17, 18]. These studies exemplify the rich and still largely undiscovered diversity of glacial environments, especially among Microbotryomycetes (Pucciniomycotina). This class contains ‘anther smuts’, formerly classified in Ustilaginomycotina, and numerous anamorphic yeasts (reviewed in [26]). It contains the orders Microbotryales (predominantly teliospore-forming plant parasites), Kriegeriales (plant parasites, aquatic fungi or saprobes from cold, temperate and tropical environments), Sporidiobolales, Leucosporidiales (anamorphic or teliospore-forming yeasts isolated from various habitats and surfaces), Heterogastridiales (filamentous fungi isolated from decaying plant material and mushrooms), Rosettozymales (yeasts from phyllosphere) and Heitmaniales (yeasts from phyllosphere) [19]. The description of the ballistospore-forming yeast from neotropical forests, *Meredithblackwellia eburnea*, and its relatedness to *Kriegeria eriophori* and *Camptobasidium hydrophilum*, led to the erection of the order Kriegeriales for the families Kriegeriaceae and Camptobasidiaceae. The taxonomic position of numerous species of the Kriegeriales could not be classified on the family level and above because their phylogenetic relationships were studied on the level of ribosomal genes only (e.g. [17, 18]), which is problematic due to low variability in LSU and high variability in ITS sequences [27]. Although the recent approaches employed multi-gene analyses [19, 27, 28], the taxonomic position of many taxa in the Microbotryomycetes remains unresolved.

Sampling campaigns in Greenland and Svalbard in 2017, investigating subglacial and glacial environments [15, 16], led to the isolation of 168 yeasts and yeast-like isolates, 150 of them belonging to the phylum Basidiomycota. Approximately half of these isolates could not be reliably identified at the species level. About half of the cultivable taxa were also detected through amplicon sequencing of the fungal internal transcribed spacer 2 (ITS2) [15, 16]. In an effort to continue the characterization of yeasts from Greenland and Svalbard collections, a new species of *Camptobasidium*, *C. arcticum*, is described as the phylogenetic sister of the recently described *Camptobasidium gelus* [18]. A new genus and species, *Psychromyces glacialis*, is also proposed to accommodate isolates identical in ribosomal sequences to ‘*Rhodotorula svalbardensis*’. Its taxonomy and status were discussed in several studies following the application of the ‘one fungus=one name’ system [27].

## Sampling sites and isolation methods

Cryoconite, snow, supraglacial ice (clear ice), supraglacial ice dominated by dark pigmented glacier algae (dark ice) and supraglacial water samples were collected from the southwest margin of the Greenland Ice Sheet during a fieldwork campaign in July 2017 [15] (Table 1). The fieldwork site was located at around 60 km east of Kangerlussuaq and was within the so-called ‘dark zone’, characterized by extensive algal blooms [29]. Subglacial ice and glacial meltwater were collected from three polythermal glaciers (Midtre Lovénbreen, Pedersenbreen and Vestre Brøggerbreen), situated in the Kongsfjorden area, Ny-Ålesund (Svalbard, Norway) in August 2017 [16] (Table 1).

**Table 1.** Data of collected samples

Sampling location	Sample type	Sampling time
Greenland, Kangerlussuaq, Ice Sheet (67° 04′ 43″ N 49° 20′ 29″ W)	Snow	July 2017
	Dark ice	
	Clear ice	
	Supraglacial water	
Norway, Svalbard, Ny-Ålesund:		August 2017
Midtre Lovénbreen (78° 53′ 37″ N 12° 04′ 13″ E)	Subglacial ice	
Midtre Lovénbreen (78° 53′ 25″ N 12° 03′ 15″ E)	Glacial meltwater	
Vestre Brøggerbreen (78° 54′ 55″ N 11° 45′ 48″ E)	Subglacial ice	
Pedersenbreen (78° 52′ 46″ N 12° 17′ 57″ E)	Subglacial ice	

All samples were collected with sterilized tools and handled with sterile nitrile gloves to avoid contamination. Samples were collected in sterile Whirl-Pak plastic bags or sterile plastic bottles and processed within a few hours of their collection at the primary ice camp on the Greenland Ice Sheet or the NERC Arctic Research Station (Ny-Ålesund). The samples were filtered and incubated at 15 °C on six different media, listed in Table S1 (available in the online version of this article), as described by Perini *et al*. [15, 16]. A total of 150 basidiomycetous yeasts and yeast-like fungi were isolated from all the above listed samples (Svalbard and Greenland [15, 16]). The majority of the strains reported in this study (53 of 74) was isolated from media with high water activity (a_w_=1) and poor in nutrients, such as minimal medium (MM) [30] and synthetic nutrient agar (SNA) [31], both supplemented with chloramphenicol (50 mg l^−1^), and from Reasoner’s 2A (R2A) agar [32]. The remaining strains (21 of 74) were isolated from media richer in nutrients, such as dichloran rose bengal chloramphenicol agar (DRBC) [33] and media with lower a_w_ (MY10–12, DG18) [34].

All strains were deposited in the Ex Culture Collection of the Infrastructural Centre Mycosmo (MRIC UL), Slovenia (www.ex-genebank.com) at the Department of Biology, Biotechnical Faculty, University of Ljubljana, Slovenia (Table S1). Around 15 % of the strains (21 of 74) could not be revived after 3 years of preservation at −80 °C using commercial preservation kit.

The holotype of the new species from Svalbard, *Psychromyces glacialis*, was deposited in the CBS culture collection of the Westerdijk Fungal Biodiversity Institute, Utrecht, as CBS 16467.

## Phenotypic characterization

Morphological characters of pure cultures were observed on potato dextrose agar (PDA) [35], oatmeal agar (OA) [36] and corn meal agar (CMA) [37] (Dalmau plating) incubated at 15 °C. For the codes used to describe colony colour we refer to [38]. Microscopic characters were observed with Nomarski interference contrast optics on Olympus BX-51 microscope, micrographs were recorded with DP72 camera (Olympus). To test for the occurrence of teleomorphic structures in the yeast group of isolates described in this manuscript as *Camptobasidium arcticum* and identified as *C. gelus*, 14-day-old yeast cultures of selected representatives were pairwise-crossed on malt extract agar (MEA), yeast extract malt extract agar (YMA), soytone–glucose agar (SG) and water agar [39] and incubated at 15 °C for 6 months. The production of hyphae and teliospores was checked regularly.

Fermentation of d-glucose was tested in liquid medium with a 2 % (w/v) solution of sugar [39]. Assimilation of carbon and nitrogen sources were evaluated in *Camptobasidium gelus* (group 1 isolates: EXF-12745, EXF-12576, EXF-12594, EXF-13102, EXF-13103), *Camptobasidium arcticum* (group 2 isolates: EXF-12713^T^, EXF-12522, EXF-12524, EXF-12711) and *Psychromyces glacialis* (group 3 isolates: EXF-13111^T^, EXF-12886, EXF-12419, EXF-12991). The assimilation of selected C and N sources was tested in liquid media according to Kurtzman *et al*. [39]; C sources: d-glucose, d-galactose, l-sorbose, d-glucosamine, d-ribose, d-xylose, l-arabinose, d-arabinose, sucrose, maltose, α-trehalose, cellobiose, salicin, arbutin, melibiose, lactose and raffinose; N sources: nitrate, nitrite, l-lysine and cadaverine. Results were read after 1, 2 and 3 weeks of incubation at 15 °C. In addition, YT Biolog plates were used according to the manufacturer’s instructions to test the assimilation capabilities of a larger number of C sources for the above-mentioned strains of *C. gelus*, isolates of group 2 and for EXF-13111 of group 3. In short, the fungi were grown on PDA for 14 days at 15 °C. Cell aliquots were resuspended in YT fluid, adjusted to reach a turbidity transmittance of about 47 % in case of yeast strains, and unadjusted for filamentous strains. Biolog YT plates were incubated at 15 °C for up to 14 days, and the assimilation was followed by measuring the absorbance at 590 nm (A590) on a CytationI3 Imaging reader, supported by the Gen5 Microplate Reader and Imager Software (BioTek Instruments). Absorbance readings were taken daily. Measurements were compared to the negative control (water) provided on the same plate, as well as to the absorbance in each individual well immediately after inoculation. Values of absorbance on day 0 in the inoculated plates were subtracted from absorbance values on days 7 and 14, and % value towards assimilation of glucose (100 %) was calculated for all the wells with assimilation tests. Calculated values of 50–70 % according to absorbance of glucose on day 7 were interpreted as weak reactions (w), values of 70–100 % (or higher) as positive reactions. After 14 days, values between 50–70 % were interpreted as weak and delayed (wd), and values between 70–100 % (or higher) as delayed reactions. With filamentous representatives of group 3, absorbance values obtained on Tween 80 were taken as 100 % reaction, interpretation of assimilation results was performed as described above. Oxidation reactions on YT plates did not give any positive reaction, and were therefore not considered.

## Phylogeny

For phylogenetic analyses, genomic DNA of the isolates was extracted using the PrepMan Ultra reagent (Applied Biosystems) according to the manufacturer instructions. DNA of filamentous cultures was extracted after mechanical lysis in CTAB buffer according to the protocol described by Gerrits van den Ende and de Hoog [40]. DNA regions/genes used in the phylogenetic analyses were the small subunit (SSU) rDNA, partial large subunit rDNA including its D1/D2 domains (LSU), the internal transcribed spacers 1 and 2 including the 5.8S rDNA (ITS), and partial sequences of genes encoding for translation elongation factor one alpha (*TEF*), cytochrome b (*CYTB*), RNA polymerase II largest subunit (*RPB1*) and RNA polymerase II second largest subunit (*RPB2*). These were amplified and sequenced with the following primer sets: NS1/NS24, NL1/NL4, ITS1/ITS4, EF1-983F/EF1-2218R, E1M4/E2mr3, RPB1-Af/RPB1-Cr, fRPB2-5F/fRPB2-7cR, respectively [27, 41–44]. Alignments of ITS and LSU sequences of strains described in this study and of the most closely related sequences of type strains and other reference strains, found with the blast algorithm [45] in the non-redundant GenBank nucleotide database, were used for phylogenetic analyses of a concatenated dataset. Partial sequences of the four protein-coding genes, *TEF*, *CYTB*, *RPB1* and *RPB2*, were selected from available sequences of the Microbotryomycetes published by Wang *et al*. [27] and Li *et al*. [19]. Accordingly, taxa such as *Cryolevonia* and *Meredithblackwellia* were excluded from analyses. Ribosomal genes were aligned in mega7 using ClustalX [46], corrections were made by hand. Protein-coding genes were initially aligned in mega7 using ClustalX, and then manually corrected according to the amino acid translations. Introns were excluded from phylogenetic analyses. The best model of nucleotide substitution was estimated with jModelTest 2.1.10 [47] and was used as the custom model (model ‘010230’) input to PhyML 3.1 to estimate the phylogenetic trees [48]. aLRT as Chi2-based support was used for calculation of branch supports. The alpha parameter of the gamma distribution of substitution rate categories and the proportion of invariable sites were estimated by PhyML. Additionally, phylogenies were reconstructed from the same alignments using MrBayes 3.2.7 [49]. Two substitution types of the 4by4 model and gamma distributed rates with a proportion of invariable sites (approximated with four categories of gamma distribution) were used for the estimation through 10 million generations (sampling every 100th generation), two runs of 15 chains each, heated at temperature 0.2 (LSU) or 0.1 (ITS, concatenated dataset) and discarding the first 25 % trees from the final consensus tree. The minimum spanning networks were constructed without ties based on a pairwise matrix of bitwise distances derived from the above described alignment of ITS using packages 'ape' and 'poppr' in R [50–52]. For the analysis of the fungal community by amplicon sequencing, the total genomic DNA was extracted in triplicate from filtered biomass and 1 g cryoconite sediment as described by Perini *et al*. [15, 16]. The PCR amplification of the ITS2 region was performed using the primers ITS4-Fun and 5.8S-Fun [53] in three reactions per sample. The sequencing of the fungal amplicons was performed by Illumina MiSeq version 3. The sequencing data were processed in QIIME2 [54] as described by Perini *et al*. [15, 16]. Among the set of representative sequences of individual amplicon sequence variants (ASVs) found in the samples, the ones similar to *Camptobasidium arcticum*, *C. gelus* and *Psychromyces glacialis* were identified with stand-alone blast [45]. These sequences were added to the set of sequences recovered from pure cultures and from the databases and aligned in mega7 using ClustalX [46]. The maximum-likelihood tree was estimated using this alignment with PhyML as described above in order to confirm the taxonomic placement of the *Camptobasidium* and *Psychromyces* ASVs.

blast searches using ITS and LSU as queries suggested that 32 isolates from our study are identical to *Camptobasidium gelus* (Pucciniomycotina, Microbotryomycetes, Kriegeriales, Camptobasidiaceae). Twenty-two isolates (group 2) could not be identified to the species level: 21 isolates differed from *C. gelus* in five positions in LSU and 17 positions in ITS, while EXF-12685, differed from *C. gelus* in 15 LSU and 45 ITS positions. Sequences of group 3 were almost identical (two and five positions difference in ITS and LSU) to ‘*Rhodotorula svalbardensis*’, described here as *Psychromyces glacialis* (Table 2).

**Table 2.** Nucleotide substitutions in ITS and LSU sequences in type strains of *Camptobasidium* species, *Psychromyces glacialis* and selected closely related species of genera *Cryolevonia*, *Glaciozyma*, *Phenoliferia*, *Rhodotorula* and *Oberwinklerozyma* Strains: 1, *Cr. schafbergensis* PYCC 8347^T^; 2, *Cr. giraudoae* CRUB 2086^T^;3, *C. hydrophilum* CCM 8060^T^; 4, *C. gelus* CBS 8941^T^; 5, *C. gelus* EXF-12745; 6, *C. arcticum* EXF-12713^T^; 7, *Camptobasidium* sp. EXF-12685; 8, *Ps. glacialis* EXF-13111^T^; 9, *G. antarctica* CBS5942^T^; 10, *G. watsonii* CBS10986^T^; 11, *G. martinii* CBS 10620^T^; 12, *Ph. psychrophenolica*. CBS 10438^T^;13, ‘*R. svalbardensis*’ MBL-I; 14, *O. yarrowii* CBS 7417^T^.Values above the diagonal are numbers of nucleotide substitutions and sequence similarity (%, in parentheses) in the LSU. Values below the diagonal are numbers of nucleotide substitutions and sequence similarity (%, in parentheses) in the sequences of the ITS.

Species	1	2	3	4	5	6	7	8	9	10	11	12	13	14
**1**		4 (99.2)	10 (97.9)	4 (99.2)	4 (99.2)	7 (98.5)	12 (97.5)	22 (95.4)	19 (96.0)	20 (95.8)	25 (94.8)	13 (97.3)	22 (95.4)	22 (95.2)
**2**	14 (97.3)		17 (96.9)	12 (97.9)	12 (97.9)	15 (97.4)	19 (96.7)	30 (94.4)	26 (95.5)	28 (95.1)	35 (93.7)	15 (97.4)	29 (94.9)	26 (95.3)
**3**	53 (88.8)	65 (87.0)		8 (98.5)	8 (98.5)	12 (97.8)	11 (98.0)	26 (95.1)	26 (95.2)	28 (94.9)	30 (94.3)	18 (96.7)	25 (95.4)	27 (94.9)
**4**	53 (88.9)	71 (86.1)	26 (94.9)		0 (100)	5 (99.1)	15 (97.4)	30 (94.4)	27 (95.2)	29 (94.9)	33 (94.1)	18 (96.9)	28 (95.1)	24 (95.7)
**5**	53 (88.9)	71 (86.1)	26 (94.9)	0 (100)		5 (99.1)	15 (97.4)	30 (94.4)	27 (95.3)	29 (94.9)	33 (94.1)	18 (96.9)	28 (95.1)	24 (95.7)
**6**	49 (89.7)	67 (86.8)	16 (96.8)	17 (96.8)	17 (96.8)		14 (97.6)	35 (93.5)	26 (95.5)	28 (95.1)	32 (94.2)	19 (96.7)	33 (94.3)	29 (94.8)
**7**	55 (88.7)	68 (86.7)	40 (91.9)	45 (91.3)	45 (91.3)	40 (92.2)		31 (94.2)	28 (95.1)	26 (95.5)	36 (93.5)	21 (96.3)	29 (94.9)	35 (93.7)
**8**	54 (88.5)	71 (85.6)	56 (88.6)	57 (89.0)	57 (89.0)	54 (89.5)	50 (90.2)		33 (93.1)	33 (93.9)	40 (92.3)	22 (95.9)	**2** (**99.6**)	32 (93.9)
**9**	67 (86.1)	81 (84.0)	69 (85.9)	81 (84.3)	81 (84.3)	77 (85.09	81 (84.3)	83 (84.1)		7 (98.8)	10 (98.2)	24 (95.8)	32 (94.4)	31 (94.4)
**10**	61 (87.2)	77 (84.7)	75 (84.8)	88 (83.1)	88 (83.1)	81 (84.4)	81 (84.3)	79 (84.9)	40 (92.6)		14 (97.5)	26 (95.5)	32 (94.4)	33 (94.1)
**11**	61 (87.0)	77 (84.4)	77 (84.2)	82 (84.0)	82 (84.0)	81 (84.1)	67 (86.9)	78 (84.9)	47 (91.1)	52 (90.2)		33 (94.1)	40 (92.8)	36 (93.3)
**12**	65 (86.2)	81 (83.6)	70 (85.6)	71 (86.1)	71 (86.1)	67 (86.8)	70 (86.4)	49 (90.4)	82 (84.0)	94 (81.8)	89 (82.5)		20 (96.5)	20 (96.4)
**13**	52 (88.9)	69 (86.0)	53 (89.2)	56 (89.2)	56 (89.2)	50 (90.3)	49 (90.4)	5 (99.1)	79 (84.9)	76 (85.5)	75 (85.4)	46 (91.0)		30 (94.6)
**14**	93 (81.0)	107 (79.2)	69 (86.1)	73 (86.0)	73 (86.0)	69 (86.7)	73 (85.9)	64 (87.5)	82 (84.0)	90 (82.6)	83 (83.7)	74 (85.6)	61 (88.1)	

**Fig. 1.**
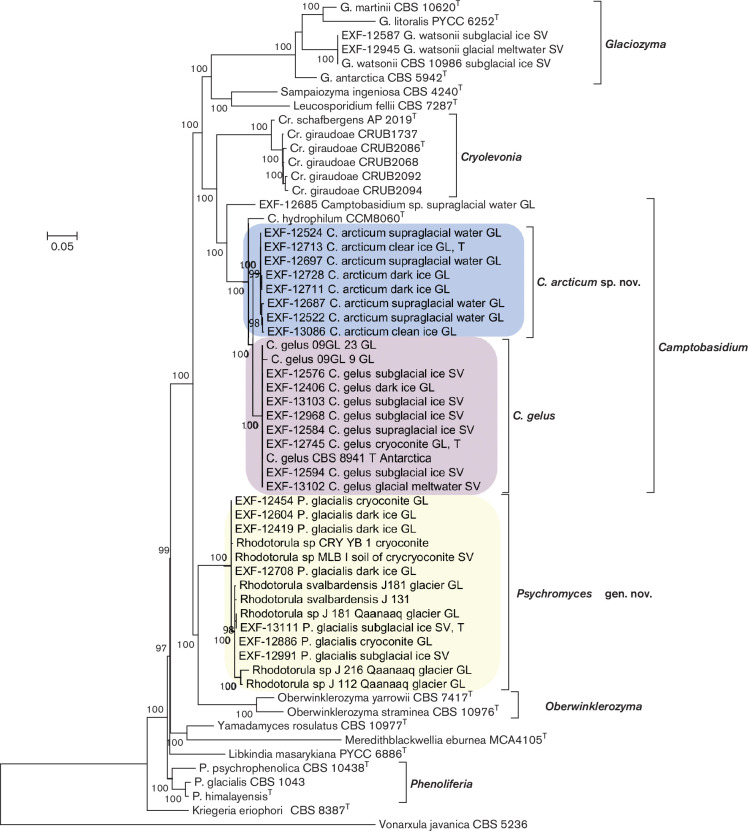
Phylogenetic tree based on alignment of the LSU rDNA and complete ITS including 5.8S rDNA, estimated by maximum likelihood. The best model of nucleotide substitution was estimated with jModelTest, other parameters (alpha parameter of the gamma distribution of substitution rate categories, proportion of invariable sites) were estimated within PhyML. aLRT as Chi2 based support was used for calculation of branch supports.

Phylogenetic analyses of concatenated sequences of the ITS and the LSU placed representatives of the newly sampled strains into well-separated lineages of the Microbotryomycetes. Group 1 isolates were identified as *Camptobasidum gelus*, recently described based on one strain isolated from Antarctica and two from Greenland [18]. Phylogenetic sister group relatedness of group 2 isolates and *C. gelus* was highly supported (branch support BS=100 %). The phylogenetic analysis confirmed that group 3 isolates belong to ‘*Rhodotorula svalbardensis*’/ *Psychromyces glacialis*. They clustered together with other sequences identified as *Rhodotorula* species. As indicated in Fig. 1, genera *Glaciozyma* (grouping together with *Sampaiozyma* and *Leucosporidium*), *Camptobasidium* and *Cryolevonia* form a highly supported monophyletic group (BS=100 %) that is a sister of a joint clade accommodating *Psychromyces* and *Oberwinklerozyma*. Genera such as *Phenoliferia*, *Yamadamyces* and *Meredithblackwellia* formed an unresolved, paraphyletic assemblage near the base of the tree, but their relatedness with *Oberwinklerozyma*, *Psychromyces*, *Camptobasidum*, *Cryolevonia* and *Glaciozyma* was highly supported (BS=100 %).

**Fig. 2.**
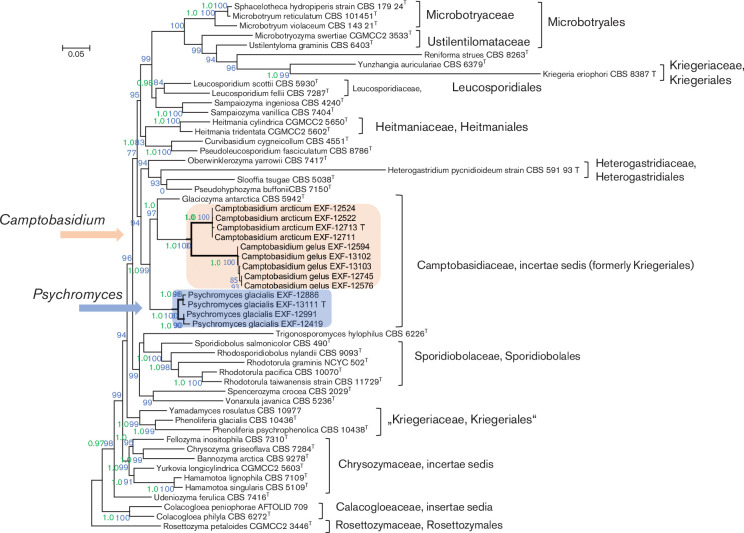
Phylogenetic tree based on alignment of the SSU, LSU, 5.8S rDNA, *TEF*, *CYTB*, *RPB1* and *RPB2* estimated by maximum-likelihood and Bayesian analyses. The tree includes representatives of *Psychromyces glacialis* gen. nov., sp. nov., *Camptobasidium arcticum sp.* nov., *C. gelus* and representative species of genera of Microbotryomycetes. In the maximum-likelihood estimation, the best model of nucleotide substitution was estimated with jModelTest, other parameters (alpha parameter of the gamma distribution of substitution rate categories, proportion of invariable sites) were estimated within PhyML. aLRT as Chi2-based support was used for calculation of branch supports. In estimation by MrBayes, a total 10 million generations were calculated and the first 25 % were discarded before the shown consensus tree was calculated from trees sampled every 100 generations. Posterior probabilities higher than 0.9 (in green) and maximum-likelihood bootstrap value from 1000 bootstrap replicates larger than 70 % (in blue) are shown near the nodes.

Further phylogenetic analyses were based on concatenated sequences of rDNA loci (SSU, ITS, LSU) and partial sequences of the four protein-encoding genes (*TEF*, *CYTB*, *RPB1* and *RPB2*). The complete alignment consisted of 9938 nt. Group 1 introns encountered in SSU sequences and introns in protein encoding genes were excluded from further analyses. The 5297 nucleotides long alignment used for analyses consisted of 1696 nt from SSU, 590 from LSU, 161 from 5.8S rDNA, 372 from *CYTB*, 746 from *TEF*, 722 from *RPB1* and 1010 from *RPB2*. Two group 1 introns (covering positions 1514–1992 in EXF-12711, 12522 and positions 2256–2690 in strains EXF-12524, 12713, 12711 of the compiled alignment) were encountered in SSU sequences of *C. arcticum. Camptobasidium gelus* and *C. arcticum* had three introns in *TEF* sequences, while there were only two in *Psychromyces glacialis*. No introns were encountered in *RPB2* and *CYTB* sequences. *CYTB* sequences could not be amplified for *C. gelus*, and *RPB1* not for *P. glacialis*. The obtained phylogenetic tree (Fig. 2) confirmed close relatedness of *Camptobasidium* and *Psychromyces* that clustered together with *Glaciozyma* (BS=99 %; BI-PP, 1.00). We therefore propose inclusion of *Psychromyces* into the Camptobasidiaceae. The analyses suggested that Kriegeriales *sensu stricto*, represented by *Kriegeria eriophori*, is relatively closely related to *Psychromyces*, *Camptobasidum*, *Glaciozyma* and others, while taxa formerly included in the Kriegeriales, such as *Phenoliferia* and *Yamadamyces*, appeared distantly related (Fig. 2). Monophyly of *Psychromyces*, *Camptobasidum*, *Glaciozyma* and *K. eriophori* is, however, not supported due to the phylogenetic interference of orders, such as Heitmaniales, Leucosporidiales and Heterogastridiales. Accordingly, classification of *Psychromyces*, *Camptobasidum*, *Glaciozyma* in the Kriegeriales is not supported in the present study, which is why the Camptobasidiaceae is for the time being considered as an *incertae sedis* within the Mycobotryomycetes (Fig. 2). It is to be emphasized, however, that the downloaded amino acid sequences of *K. eriophori* (CBS 8387) were partly difficult to align with numerous other included taxa (*RPB1*, *RPB2*) and that several codons could not be translated into amino acids at all (*CYTB*). It is clear that these numerous apomorphies are responsible for the long terminal branch that *K. eriophori* obtained in analyses of the non-translated DNA sequences (Fig. 2).

To analyse the effect of protein-encoding gene sequences on the dataset analysed in Fig. 2 and to allow comparison with already published trees that are based only on nc rDNA data, protein-encoding genes were excluded from the dataset and the remaining alignment was analysed separately (Fig. S1). This analysis also addressed more Mycobotryomycetes as included in Fig. 1 and supported some of the conclusions made on the basis of concatenated dataset presented in Fig. 2. Relatedness of *Camptobasidium*, *Psychromyces* and *Glaciozyma* is almost equally highly supported (BS=92 %), while the monophyly of the so defined Camptobasidiaceae with *Kriegeria sensu stricto* is not supported. Monophyly of *Kriegeria eriophori* with *Phenoliferia* and *Yamadamyces* (Fig. S1, BS=89 %) is, however, not in congruence with inferences made on the basis of the dataset including protein-encoding genes (Fig. 2). The inconsistent clustering of *K. eriophori* is most likely caused by numerous apomorphies that resulted in the above-discussed alignment problems and a long terminal branch for *K. eriophori* in Fig. 2.

## Camptobasidium

**Fig. 3.**
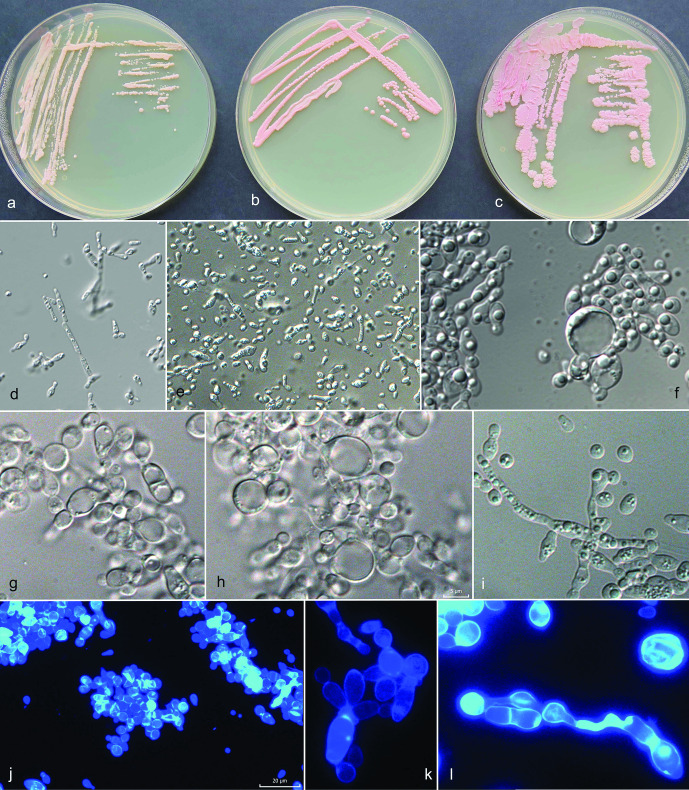
Morphology of *Camptobasidium arcticum*. (a–c) Cultures of *Camptobasidium arcticum* on PDA in 9 cm Petri dishes after 2 months of incubation at 15 °C: (a) EXF-12713^T^, (b) EXF-12689, (c) EXF-13086. Micromorphology of cells in water: (d) EXF-12689 on PDA after 14 days, (e) after 2 months, (f) EXF-12713^T^ on PDA after 14 days, (g, h) on OA after 2 months, (i) EXF-12689 on OA forming pseudohyphae, (j–l) EXF-12713^T^ on OA stained with calcofluor white. Scale bar indicated on fig. (j) (20 µm) is valid also for figures (d) and (e) — ×400 magnification; scale bar indicated on fig. (h) (5 µm) is valid also for figures (f), (g), (i), (k), (l) – ×1000 magnification.

**Fig. 4.**
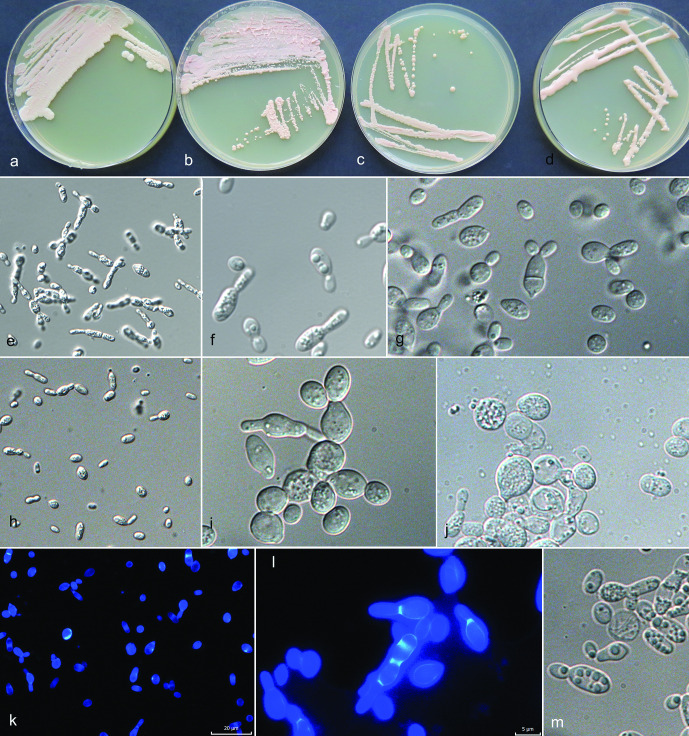
Morphology of *Camptobasidium gelus*. (a)–(d) Cultures of *C. gelus* on PDA in 9 cm Petri dishes after 2 months of incubation at 15 °C. (a) EXF-12597, (b) EXF-13102, (c) EXF-12968, (d) EXF-12745. Micromorphology of cells in water, (e, f) EXF-12745 on PDA, 14 days, (g) EXF-12745 on OA, 28 days, (h) EXF-12968 on PDA and (i) on OA, (j) EXF-12576 on PDA, (k, l) EXF-12745 on OA stained with calcofluor white. (k) EXF-12586 on PDA. Scale bar indicated on fig. (k) (20 µm) is valid also for figures (e) and (h) – ×400 magnification; scale bar indicated on fig. (l) (5 µm) is valid also for figures (f), (g), (i), (j), (l), (m) – ×1000 magnification.

The maximum-likelihood analysis of selected ITS and LSU sequences of *Camptobasidium* strains supported the recognition of three species: 31 strains were identified as *C. gelus* and 22 strains as two yet undescribed species, of which one (EXF-12685) is no longer alive. The other species is here newly described as *C. arcticum*. The ITS/LSU sequences of *C. arcticum* strain EXF-12713 differed from the those of *C. gelus* CBS 8941 by 17/5 nt (99/96,9 % similarity). Strain EXF-12685 was most closely related to *C. hydrophilum* CCM8060 (11 nt difference in LSU, 98 % similarity; 40 nt difference in ITS, 91.9 % similarity), while it differed from *C. arcticum* strain EXF-12713 by 14 nt in the LSU and 42 nt in the ITS sequences. A similar comparison with *C. gelus* strain EXF-12745 showed a difference of 2.6 % (15 nt) in LSU, and 8.6 % (45 nt) in ITS (Table 2). Since EXF-12685 did not survive deep-freezing preservation, it could not be taxonomically considered. The representatives of other closely related species listed in Table 2 had lower similarities and the number of differing nucleotides in ITS was for all drastically larger as in LSU, as already described in other studies [27]. When comparing ITS sequences almost no variability was noted in *C. gelus*, while some were noted within *C. arcticum* (Fig. S2). The type strain of *C. gelus*, CBS 8941 (AY040665) from Antarctica, strain BL58-2 (AB474396) isolated from Russian glacier ice core in Siberia [55] and EXF-12745 from cryoconite in Greenland, had identical ITS sequences. The Antarctic yeast strain CBS 8941, now recognized as *C. gelus*, was noticed as a potentially new phylogenetic lineage already by Wang *et al*. [23] on the level of LSU sequences. It was then selected as the type of *C. gelus* by de Garcia *et al*. [11], who described this species based on two additional strains from Greenland. The same authors [23] addressed the separate position of CRUB 1733 (GenBank FJ841888), which was recently described as the new species *Cryolevonia giraudoae* by de Garcia *et al*. [11]. The genus *Cryolevonia*, closely related to *Camptobasidium* (Fig. 2), was described and typified with *Cr. schafbergensis* by Pontes *et al*. [17]. Classification of these psychrophilic yeast species was based only on ITS /LSU data [11, 17]. The type species of the genus *Camptobasidium*, *C. hydrophilum,* is strictly filamentous and forms peculiar anamorphic Ingoldian spores [56], and no yeast phase, which is the only morphology observed in *C. gelus*. Analyses of ribosomal DNA sequences placed two of our three newly discovered phylogenetic groups in *Camptobasidium*; however, ribosomal DNA sequences are only restrictively used for phylogenetic analyses of the Microbotryomycetes [23]. No household gene sequences could be generated or are accessible for the type species, *C. hydrophilum*, which is why monophyly of all *Camptobasidium* species cannot be confirmed yet on the basis of all loci selected here. *Camptobasidium* cultures are characterized by pink to reddish-grey colonies [11, 56], in our case studied on PDA (Figs 3 and 4). According to our observation, strains identified as *C. gelus* were typically purplish white (14A2), pastel pink (11A3), greyish rose (11B3) or reddish grey (11B2), with either glistening or ridged, membranous surface appearance, raised with entire or undulating margin (Fig. 4a–d). De Garcia *et al*. [11] described *Camptobasidium gelus* as ovoidal to ellipsoidal yeasts multiplying by multilateral budding. According to the provided micrograph [11], they also observed larger cells containing large vacuoles, as well as smaller storage granules, which were observed also in our isolates (Fig. 4f, j and m). The original description of *C. gelus* states that hyphae or pseudohyphae are not formed, however we observed the presence of weak pseudomycelium on PDA and in Dalmau plate culture on CMA in our isolates (Fig. 4e, l). Individual yeast cells of *C. gelus* isolates from this study were oblong, 5.5±2 (mean±SD; min-max: 3–9)×3.5±1 (mean±SD; min-max: 2–5) µm when one-celled, up to 13 µm long when two-celled, while *C. arcticum* forms slightly smaller cells, measuring 5±2 (mean±SD; min-max: 3–9)×3±0.6 (mean±SD; min-max: 2–4) µm due to developed pseudomycelium less glistening colonies.

Sexual reproductive structures were neither observed in single colonies or in mixed cell assays of *C. gelus*, nor in *C. arcticum*. No true mycelium and no teliospores were observed. The sexual morph is only known for *C. hydrophilum* from water-submerged cultures and is characterized by recurved, 1–3 septate metabasidia showing sporogenous loci on their convex sides and bearing rarely single basidiospores, mostly in groups of up to 20 that are sessile on inconspicuous denticles [56]. Authors also reported typically broadly fusiform or elliptical chlamydospores.

All *Camptobasidium* strains in our study showed slow colony development suggesting either oligotrophicity or auxotrophy, thus perhaps the need of a particular nutrient, mediating substance or partner relationship. Also, the closely related and psychrophilic *Cryolevonia schafbergensis* was described as a species with salient physiological characteristics: not growing at 18 °C, growing on a medium containing 16 % NaCl. These characteristics allowed the distinction between *C. giraudoae* [11] and *Camptobasidium* species (Table S2).

Differences in assimilation profile among closely related species are listed in Table 3, and within *Camptobasidium* and *Psychromyces* in Table S2. Assimilation of raffinose and sucrose was evident in all *Camptobasidium* species, but also in *Psychromyces glacialis*. Assimilation abilities of *C. gelu*s isolates were mostly in accordance with de Garcia *et al*. [11]. Our isolates variably assimilated l-sorbose, d-xylose and nitrite, but were not able to assimilate melibiose and polyols (i-erythritol, xylitol, d-mannitol, d-sorbitol, adonitol) (Table S2). The newly proposed *C. arcticum* differs from *C. gelus* in the ability to assimilate melezitose, cellobiose, methyl β-d-glucoside and in the inability to assimilate nitrite. The additional differences in assimilation of other, not often used C sources were noticed in *C. arcticum*: the ability to assimilate gentiobiose, maltotriose and dextrin. It variably assimilated nitrate and showed weak or no assimilation of l-lysine. In some cases, differences in assimilation were observed when comparing results of classical tests in liquid media with the results of Biolog YT. As an example, *Camptobasidium gelus* showed assimilation of maltose and glycerol in classical tests, but no optical density changes were detected on YT plates (Table S2).

**Table 3.** Differences and similarities in the assimilation capacity of selected carbon and nitrogen sources among *Glaciozyma*, *Cryolevonia*, *Camptobasidium* and *Psychromyces* species Species: 1, *Glaciozyma antarctica* [62]; 2,*Glaciozyma watsonii* [24]; 3, *Glaciozyma martini* [24]; 4, *Glaciozyma litorale* [25]; 5, *Cryolevonia schlafbergensis* [17]; 6, *Cryolevonia giraudoae* [11]; 7, *Camptobasidium hydrophilum* [11]; 8, *Camptobasidium gelus* [11]; 9, *Camptobasidium gelus* (this study); 10, *Camptobasidium arcticum* (this study); 11, *Psychromyces glacialis–*yeast phase (this study); 12, *Psychromyces glacialis–*filamentous phase (this study); 13, ‘*Rhodotorula svalbardensis*' [57]. +, Positive; –, negative; w, weak positive; d, delayed (slow); wd, weak and delayed; v, variable (–/+/w/d); na, data not available.

Source	1	2	3	4	5	6	7	8	9	10	11	12	13
l-Arabinose	–	v	w/–	–	–	–	w	–	–	–	–	–	–
d-Arabinose	–	–	–	–	–	–	w	–	–	–	–	–	+
α-d-Glucose	+	+	+	+	+	+	+	+	+	+	+	+	+
d-Galactose	v	+	+	–	–	v	na	v	v (–,w,d)	–	d	–	–
Sucrose	v	–	–	–	+	–	+	+	+	+	+	+	+
Raffinose	–	–	–	–	–	v	+	+	+	d	+	+, –*, d	+
d-Ribose	–	v	–	–	–	–	na	–	v (d,w,+)	–	–	–	–
d-Sorbitol	v	+	w/+	na	na	w	na	+	–*	–*	wd*	–*	na
Lactose	–	–	w/–	–	–	–	+	–	w/–	v	–	–	+
Trehalose	v	w/–	w/–	v,d	–	–	–	–	v	w/+	+	d	na
Maltose	v	v	v	–	+	v	w	w	w/+, –*	+	d	+, w*, d	+
Melezitose	–	–	–	–	+	+	+	–	–	+	+	+	+
Melibiose	–	–	–	–	–	–	–	+	–	v, –*	–	–	–
Methyl α-d-glucoside	–	–	–	–	–	–	w	–	–*	–*	–*	–*	na
d-Xylose	v	v	w/–	–	–	–		–	w/d	v (–,w,d)	wd	–	–
d-Glucitol	v	na	w/+	–	v	na	na	+	–*	–*	wd*	–*	na
Cellobiose	v	–	–	v,d	–	w	na	w	–	+	w,–*	w/+	+
d-Glucosamine	na	–	–	–	–	–	na	–	–*	–*	–*	–*	na
Glycerol	v	–	v	d	d	+	–	+	v, –*	–	–	–	–
Nitrate	+	w/+	+	na	+	+	–	+	+	v	+	+	+
Nitrite	+	–	w	na	+	+	–	+	w/–	–	+	w/d	+
l-Lysine	na	v	d/–	na	–	+	na	d	+	w/–	w	w/–	na
Tween 80	na	na	na	na	na	na	na	na	v (–,w,+)	–	+	+	na

*Results obtained by Biolog YT plates only.

## Psychromyces

The maximum-likelihood analysis of selected ITS and LSU sequences grouped together dimorphic and filamentous isolates of group 3 with isolates named *Rhodotorula* sp. and *Rhodotorula svalbardensis* that were all isolated from cold environments of Svalbard and Greenland [12, 57]. The strongly supported sister group relationship of this group 3 to *Oberwinklerozyma* was suggested on the basis of analyses of nc rDNA sequences (BS=100 %) (Fig. 1). The concatenated seven-gene analysis justified and confirmed the isolated position of the genus (BS=100 %), and placed it as sister clade to the clade comprising *Camptobasidium* and *Glaciozyma* (Fig. 2). These results suggest that the clade should be placed in the family Camptobasidiaceae. We propose here a new genus, *Psychromyces*, for this clade for reasons described below. Bayesian phylogenetic inference analysis based on ITS sequences revealed this clade as unresolved (Fig. S2). A number of six nucleotide substitutions out of 526 distinguish two groups of strains, represented by EXF-13111 and EXF-12419 (Fig. S5). Exploration of the clade with additional phylogenetic marker genes (*CYTB*, *TEF*, *RPB2*) did not result in any better phylogenetic resolution. Accordingly, only a single species, *P. glacialis,* could be recognized. Strains with highly similar or identical ITS and LSU sequences were found also in other studies of Arctic glacial environments and supported cladification into two phylogenetically unresolved or paraphyletic subgroups within *Psychromyces*. Strains EXF-13111, 126476, 12545, 12886, 12991 belong to one, and strains EXF-12419, 12623, 12398, 12718, 13156, 124554, 12890, 12708, 13154, 12420, 12604, 12984, 12626 to the other group (Figs S2b and S5). Strains from Qaanaaq (represented by KY782280, KY782283) and Russell glaciers (KY782281, KY782282), both from Greenland (Singh *et al*., unpublished), and a strain from cryoconite sediment from Midtre Lovénbreen glacier in Svalbard (KC333170) [12] clustered with EXF-13111 on the basis of ITS sequences. The second group is represented by strain EXF-12419. Its ITS sequence is almost identical to the one of the strains MLB-I (JF805370) and CBS 12863 (=JCM 19699, JCM 19700, MTCC 10952) (AB734690), of which the latter originated from cryoconite soil sampled from the Midtre Lovénbreen glacier, Svalbard [57].

Singh *et al*. [57] erected the name ‘*Rhodotorula svalbardensis’* for strains MLB-I and CBS 12863; however, the name *Rhodotorula svalbardensis* is invalid because two gatherings, strains MLB-I (CCP-II) and CRY-YB-1, were assigned as type [58], Shenzhen Code, Art. 40.7). Colony characteristics and microscopic characters described by Singh *et al*. [57] clearly suggested conspecificity of strains MLB-I (CCP-II), CRY-YB-1 and several strains we obtained from Svalbard and Greenland. However, although our strains were isolated as yeast colonies, only a single strain, EXF-13111, retained its ability to grow as a yeast after 2 years of preservation at −80 °C, while the other 16 strains were revived as purely filamentous after deep freezing. Singh *et al*. [57] described and illustrated ellipsoidal vegetative cells, 5.8–8.3×4.2–7.5 µm (mean 7×5.9 µm), occurring individually or in groups. According to our observation, these thick-walled cells most likely represent teliospores. In our strains these structures were typically globose to subglobose and had a diameter of 3–4 µm in young cultures; however, depending on the culture condition and age of culture, also spores with a diameter of up to 11 µm and of variable shapes were seen. Sometimes they contained oil droplets. In an attempt to induce their germination, the spores were cut out, placed into sterile H_2_O and kept at 5 °C. After an incubation period of 6 months, they were placed on water agar, however, no germination was observed. Chlamydospores are known in some species of basidiomycetous yeasts, for example, in genera *Mrakia*, *Sporidiobolus*, *Tilletiaria*, *Fellomyces* and *Rhodosporidium*, where they can appear in old cultures [59]. They were also observed in *Camptobasidium hydrophylum* [56]. Singh *et al*. [57] described, however, not illustrated, unilaterally or occasionally multilaterally budding cells. Yeast colonies were observed only in the strain EXF-13111. The yeast phase was maintained with the subculturing of yeast colonies, however after approx. 1 month of incubation, the colonies started developing hyphae and became strictly filamentous. The observed yeast cells were oblong and measured 7.5±3 (mean±SD; min-max: 5–12) µm×3.5±0.5 (mean±SD; min-max: 3–4) µm. Budding was uni-, bi- or multilateral and occurred solitarily directly on the mother cell or on sympodially proliferating, up to 5 µm long stalks. Daughter yeasts formed on terminal or lateral sympodially proliferating stalks have been so far described for certain *Bullera* species [59]. Some of the yeast cells in EXF-13111 prolonged to more than 20 µm long stalks, and formed pseudomycelium-like structures. Strains described by Singh *et al*. [57] formed up to 1.45 µm wide, septate hyphae. The presence of hyphae, with and without clamps, was observed in some strains in our study. Typically, the width of clamped mycelium was 2.5 µm in strain EXF-12419, while hyphae without clamps were narrower (up to 2 µm) and densely septated. In strain EXF-13111, non-clamped mycelium had a width of approx. 1.5 µm. If filamentous colonies were subcultured, they remained filamentous.

The most obvious difference between *Psychromyces* and other related species was its ability to assimilate Tween 80 (reaction on YT plate), which indicates lipolytic capacity. *Camptobasidium gelus* isolates varied in assimilation of Tween 80 (negative, positive, week reactions), while *C. arcticum* was unable to assimilate it. The assimilation ability of Tween 80 is in agreement with Singh’s *et al*. [57] observation of high lipase activity. In contrast, amylase activity reported by the same authors [57] was not detected by the classical assimilation tests performed in this study. The assimilation profile was similar to the closely related genus *Camptobasidium*: sucrose and raffinose were assimilated, as for all species of *Camptobasidium*; however, *Psychromyces* showed also the ability assimilate trehalose (Tables 3 and S2). It assimilated melesitoze and cellobiose, as *C. arcticum*. According to Singh *et al*. [57], *Psychromyces glacialis* is well-adapted to life in glacial environments, in particular to low temperatures. It can synthesize antifreeze proteins, modulate its membrane lipid composition by increasing the unsaturated fatty acids content and produce extracellular enzymes, particularly amylase, cellulase, protease and catalase at 4 °C [57].

## Ecology

Ice samples retrieved from the Greenland Ice Sheet harboured a high abundance of yeasts, e.g. up to 900 c.f.u. ml^−1^ in dark ice, and contained a large number of yeast taxa. The majority of the taxa belonged to the Microbotryomycetes (Basidiomycota), including *Camptobasidium gelus*, the here proposed *C. arcticum*, formerly classified as *Glaciozyma antarctica*-like, *Psychromyces glacialis*, formerly classified as ‘*Rhodotorula svalbardensis’*, *Phenoliferia glacialis*, *Sporobolomyces ruberrimus*, and other yet-undescribed basidiomycetous yeasts [15]. A study of glacial environments on Svalbard resulted in the isolation of some of the same taxa, such as *C. gelus* and *Psychromyces glacialis*, but also revealed additional species: *Glaciozyma watsonii*, *Leucosporidiella muscorum*, *Phenoliferia glacialis* and *P. psychrophenolica*, with values up to 5 c.f.u. ml^−1^ [16]. The phylogenetic and phenotypic analysis of both sets of glacial isolates supported the description of two novel species, herewith proposed as *Camptobasidium arcticum* and *Psychromyces glacialis*.

High-throughput amplicon sequencing and analysis of ITS2 sequences from total environmental DNA [15, 16] revealed that species of the Microbotryomycetes commonly and abundantly occur in supra- and subglacial environments. The abundance of this group was up to 96 % in supraglacial water, and clear and dark ice (Greenland Ice Sheet) and up to 75 % in subglacial ice (Svalbard). ITS2 sequences identical to those of *Camptobasidium gelus* EXF-12745, *Camptobasidium arcticum* EXF-12713, *Camptobasidium* sp. EXF-12685 and *Psychromyces glacialis* EXF-13111 were found in total environmental DNA extracts (Fig. S3). Accordingly, these species appeared to commonly occur in diverse glacial environments and different geographical locations. The genus *Camptobasidium* has a circumpolar distribution and it also occurs in Antarctica [18]. The genus *Psychromyces* has so far only been recorded in Arctic glacial environments, both in Greenland and Svalbard. None of the species has been found so far in uppermost layers of the glacier consisting of fresh snow.

Slow growth of axenic cultures and unsuccessful preservation of circa 15 % of the isolates might indicate that *Camptobasidium* species, especially *C. arcticum*, accommodates a complex ecological niche or that it has complex nutritional requirements. It is possible that they grow optimally only in associations with other glacial organisms. Myco- and plant-parasitism is a frequent trait of the Pucciniomycotina, including Microbotryomycetes [60]. *Camptobasidium hydrophilum*, a close relative of *C. arcticum*, was identified as a mycoparasite, since it can form coiling hyphae around hyphae of aquatic hyphomycetes in dual cultures [56]. Parasitic life mode at the host–parasite interface is otherwise known to be supported via special interactive organelles, called colacosomes. These structures were so far detected in mycoparasitic Heterogastridiales, but also, occasionally, in non-parasitic Sporidiobolales and Leucosporidiales within Microbotryomycetes [60], implying their loss in saprobic species. The presence of these structures, indicative of mycoparasitism, have not been studied in *Camptobasidium* and *Psychromyces* so far, and should be the focus of future studies.

Statistical analysis of ITS2 NGS sequencing data from Greenland glacial environments revealed high co-occurrence of non-identifiable Microbotryomycetes with *Phialophora* (Chaetothyriales, Ascomycota) and with unidentified Leucosporidiales (Mycrobotryomycetes, Basidiomycota). While in Svalbard glacial samples high co-occurrence was calculated for non-identifiable Microbotryomycetes with an unidentified species of Leucosporidiales, with Didymellaceae (Pleosporales, Ascomycota), with *Libkindia masarykiana* (incertae sedis, Microbotryomycetes), and with Kriegeriaceae (Kriegeriales, Microbotryomycetes; data not shown). These co-occurrences might indicate similar ecological preferences of these taxa (e.g. for cold aquatic environments) or interactions between the taxa.

The transition from species only showing a yeast phase, a presumable ancestral morphology to species also having a filamentous phase, co-occurs with ecological niche diversification and adaptations to various lifestyles and environments [60]. Exclusively filamentous species *in vitro* are found only in *Heterogastridium*, *Pycnopulvinus* (both Heterogastridiales), some genera of the Microbotryales, *Camptobasidium* (type species) and *Psychromyces*. It is unclear whether *C. gelus* or *C. arcticum*, showing only yeast growth *in vitro*, produce hyphae in their natural environment. However, it is rather likely that their life styles in nature are far more complex than can be predicted by *in vitro* studies. Occurrence of teliospores and clamped mycelium in *P. glacialis*, for example, implies that this species might produce basidia and basidiospores in nature, although these structures have not yet been observed on agar plates. Similarly, only a single *P. glacialis* strain displayed dimorphic characters while all other strains were strictly filamentous. Although the ecological role of this species is yet unresolved, numerous adaptations to cold environments have been already recognized [53].

This study shows that the glacial environments of Greenland and Svalbard harbour a high abundance of ecologically highly adapted species of the Microbotryomycetes. Additionally, a large yeast diversity remains uncharacterized, which should be investigated in future studies with novel isolation, cultivation and preservation approaches. These studies should also investigate species interactions, which might be particularly important in environments characterized by extremely low temperatures, lack of water and oligotrophic conditions. The presented results also underline the power of combining culture-dependent and independent strategies to assess yeast diversity and to progress towards unravelling the function of these yeasts in glacial ecosystems.

## Description of *Camptobasidium arcticum* sp. nov. (Perini & Zalar)

*Camptobasidium arcticum* (arc'ti.cum. L. neut. adj. *arcticum*, northern, Arctic, referring to the geographical area the species was discovered).

MycoBank number: MB 834188

Streak cultures at 15 °C after 14 days on PDA mucoid, reddish white (8A2, 11A2), made of minute colonies. Colonies at 15 °C after 8 weeks on PDA 2–4 mm, either pastel pink (8A2) to pink (11A5) with glistening or rough surface with a wrinkled structure, almost entire margin in the glistening colony type, margin undulate in rough colony type (Fig. 3a–c). Individual yeast cells on PDA one- or two- celled, oblong, 5±2 (mean±SD; min-max: 3–9)×3±0,6 (mean±SD; min-max: 2–4) µm, often filled with inclusions (storage granules), some cells swell to round, up to 10 µm large structures, containing a single large vacuole (Fig. 3f–h). Yeasts are occurring singly or in clusters. Budding is predominantly uni- and bi-polar (Fig. 3g), but also with several loci at one pole (Fig. 3k). Pseudohyphae observed in some strains on OA (Fig. 3l). Sexual reproductive structures not observed in single or mixed cell assays.

Assimilated compounds: α-d-glucose, sucrose, trehalose, raffinose (delayed), maltose, melezitose, cellobiose, gentiobiose, maltotriose, stachyose (delayed), methyl β-d-glucoside, dextrin (sometimes delayed) and cadaverine (sometimes weak and delayed). Variable: melibiose, lactose, salicin, palatinose, d-xylose, maltitol, nitrate and l-lysine. The following compounds are not assimilated: d-galactose, starch, l-sorbose, l-arabinose, d-arabinose, d-ribose, d-glucosamine and nitrite. Additional data on C source assimilation obtained by Biology YT plates only are given in Table S2.

Does not grow in the presence of 10 %, 15 % NaCl or 50 % glucose.

Growth at 5, 10, 15 °C is positive; no growth is evident at 20 °C. Fermentation abilities absent.

The holotype, EXF-12713H, originated from clear ice in Greenland Ice Sheet, 60 km east of Kangerlussuaq, 67° 04′ 43″ N 49° 20′ 29″ W, in July 2017 by Laura Perini. It is permanently preserved in a metabolically inactive state at the Ex Culture Collection of the Infrastructural Centre Mycosmo (MRIC UL), Slovenia (www.ex-genebank.com) at the Department of Biology, Biotechnical Faculty, University of Ljubljana, Slovenia. Accession numbers of DNA sequences derived from ex-type strain EXF-12713, deposited into the above mentioned Ex Culture Collection: MK454798 (LSU), MN983248 (ITS), MT304813 (SSU), MT260394 (*CYTB*), MT260390 (*TEF*), MT260386 (*RPB2*).

Other examined strains: Greenland, isolated from supraglacial water (EXF-12522, EXF-12524, EXF-12689), from dark ice (EXF-12711), from clear ice (EXF-13086), August 2017, L. Perini.

*Camptobasidium arcticum* (Fig. 3) differed from *C. gelus* (Fig. 4) in several phenotypic characteristics, such as colony colour and morphology, colony and cell size. Moreover, the newly proposed *C. arcticum* differs from *C. gelus* in the ability of assimilation of melezitose, cellobiose, gentiobiose, maltotriose, methyl β-d-glucoside and dextrin, in the inability of assimilation of nitrite, in variable assimilation of nitrate, and in weak or no assimilation of l-lysine. *Camptobasidium arcticum* was isolated from all sampled environments in Greenland, with no evidence of a clear preference for a specific habitat. *Camptobasidium gelus* occurred in cryoconite, dark ice, clear ice, supraglacial water in Greenland and in subglacial ice and glacial meltwater in Svalbard. None of the species were recovered from snow. *Camptobasidium arcticum* was until now found only in Greenland samples of this study. Three identical LSU rDNA sequences (JQ768846, AB558448, AY040647) were deposited at NCBI. The first sequence (JQ768846) was named Basidiomycota sp. TP-Snow-Y73 by Shao and Ma and was isolated from glacier surface snow of the Tibetan plateau (PR China), but no publication has been linked to the strain. The second sequence (AB558448), named Basidiomycota sp. GU54, was produced by Uetake *et al*. [61], and was isolated from glacial surface ice and snow of the Gulkana glacier (Alaska).

## Description of *Psychromyces* gen. nov. (Perini & Zalar)

*Psychromyces* (Psy.chro.my’ces. Gr. masc. ad. *psychros* cold; Gr. masc. n. *mykes* a fungus; N.L. masc. n. *Psychromyces* a fungus from the cold).

Belongs to phylum Basidiomycota, subphylum Pucciniomycotina, class Microbotryomycetes, family Camptobasidiaceae. The genus is circumscribed based on phylogenetic analyses shown in Figs 1 and 2, with close relationships to the genera *Camptobasidium*, *Glaciozyma* and *Cryolevonia*. Cultures pink, dimorphic or filamentous, initially yeast-like, after incubation filamentous with some or no remaining yeast colonies. Hyphae septate, clamp connections absent or present. Oblong intercalary teliospores produced after 1 month of incubation. Sexual reproduction in the form of germinating basidia from teliospores and basidiospores is not known, but clamped hyphae indicate its sexual reproduction. Assimilated C sources: α-d-glucose, sucrose, raffinose and melezitose. Assimilated N sources: nitrate, nitrite and cadaverine. Glucose is not fermented. Urease reaction is positive. Type species is *Psychromyces glacialis* (Perini & Zalar).

MycoBank accession number: MB 834189.

Type species: *Psychromyces glacialis* (Perini & Zalar).

Species accepted: *Psychromyces glacialis* (Perini & Zalar) MB 834190.

## Description of *Psychromyces glacialis* sp. nov. (Perini & Zalar)

*Psychromyces glacialis* (gla.ci.a’lis. L. masc. adj. *glacialis* icy, frozen).

MycoBank accession number: MB 834190.

**Fig. 5.**
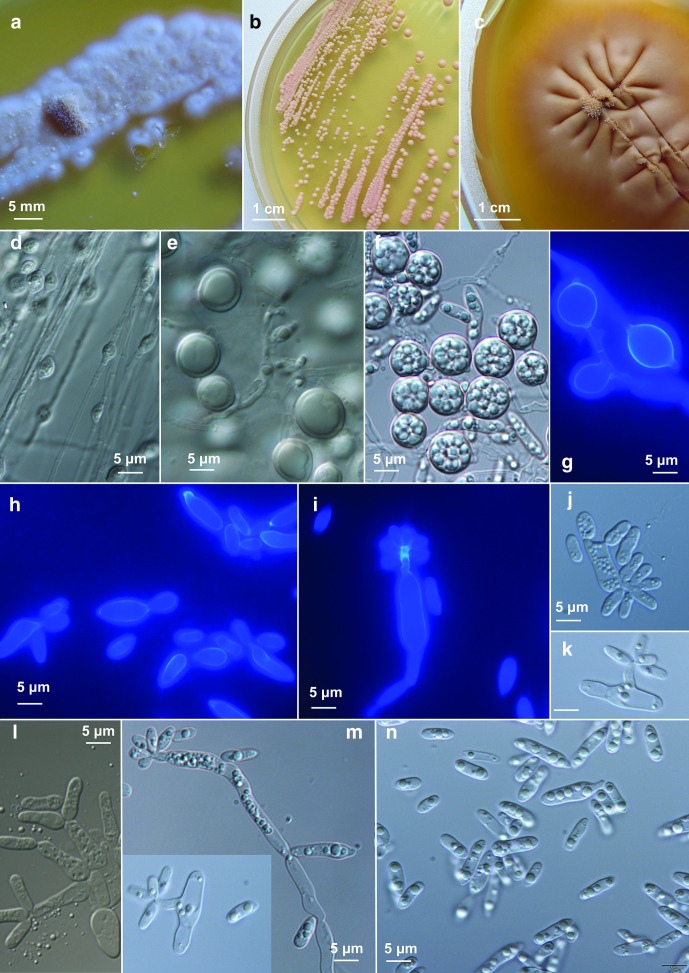
Morphology of *Psychromyces glacialis*. (a, b) cultures of *Psychromyces glacialis* EXF-13111 at 15 °C: (a) PDA, dimorphic culture after 3 weeks of incubation, (b) PDA, yeast culture grown from single yeast colony after 1 month of incubation, (c) PDA, filamentous culture grown from the margin of filamentous colony after 1 month of incubation; (d–g) teliospores grown on (d) CMA, Dalmau plate after 1 month of incubation in 60 % lactic acid, (e) PDA, margin of filamentous colony after 10 weeks of incubation in 60 % lactic acid, (f) PDA, yeast part of colony after 3 weeks of incubation, mounted in water, (g) PDA, filamentous colony after 3 weeks, coloured with calcofluor white; (h–n) yeast cells, (h, i, m, n) PDA, from dimorphic culture after 3 weeks of incubation, (h–i) mounted in water, (m–n) stained with calcofluor white (l) PDA, dimorphic culture in water after 1 month of growth on PDA, rudimentary pseudohyphae; (i–m) PDA, budding cells on short or long denticles, (n) PDA, yeast cells with inclusions.

Streak cultures at 15 °C after 14 days on PDA agar mucoid, orange white (5A2), filamentous, 2–6 mm diameter, margin composed of fine filaments immersed into the medium (Fig. 5a). Synnemata-like dense bundles of hyphae form on initial inoculation points (Fig. 5c). Yeast colonies (Fig. 5b) visible after 3 weeks, attain 2 mm diameter after 7 weeks on PDA and MEA, 0.5 mm on SNA, pastel red (7A4), convex and mat. After 3 months on PDA at 15 °C, the filamentous colonies reach up to 40 mm diameter, become pale red to pastel red (7A3, 8B4), mat. Mycelium in some strains without clamps (EXF-13111), 1.5–2.0 µm wide (Fig. 5d), or with clamps (Fig. S6), 2–4 µm wide, branched (Fig. S6 d–j). After 14 days of incubation, numerous teliospores are produced intercalary (Figs 5d–g and S6 h, j–l). Teliospores oblong, (5.5–) 8×10 (–13) (mean; min-max: 5.5–13) µm. Budding occurring in yeast colonies polar or occasionally multilateral, sessile or on short or long denticles, and with sympodial proliferation. Individual yeast cells on MEA: 8.5±2 (mean±SD; min-max: 5.5–11)×(2.5) 3.5±0.5 (4.5) µm, on PDA: 7.5±3 (mean±SD; min-max: 5–12)×3.5±0.5 (mean±SD; min-max: 3–4) µm. Dalmau plate culture on CMA agar, 7 weeks of incubation: numerous teliospores on densely septated mycelium.

Assimilated compounds: α-d-glucose, sucrose, raffinose, trehalose, maltose, melezitose, cellobiose (sometimes weak), nitrate, nitrite and cadaverine. Variable: salicin, l-sorbose, d-xylose, l-arabinose, d-glucosamine and l-lysine. Not assimilated compounds: melibiose, d-galactose, starch, d-arabinose, d-ribose and glycerol. Additional data on C source assimilation obtained by Biology YT plates only are given in Table S2. Does not grow in the presence of 10%, 15 % NaCl, 50 % glucose.

Growth at 5, 10, 15 °C is positive; no growth evident at 20 °C. Fermentation abilities absent.

The holotype, CBS 16467, originated from subglacial ice of Vestre Brøggerbreen glacier (78° 54′ 55″ N 11° 45′ 48″ E) in Norway, Svalbard, in July 2017 by Laura Perini. It is permanently preserved in a metabolically inactive state at the CBS Yeast Collection of the Westerdijk Fungal Biodiversity Institute, Utrecht, the Netherlands. The ex-type strain has been deposited in the Ex Culture Collection of the Infrastructural Centre Mycosmo (MRIC UL), Slovenia (www.ex-genebank.com) at the Department of Biology, Biotechnical Faculty, University of Ljubljana, Slovenia, as strain EXF-13111. Accession numbers of DNA sequences derived from type: MT301949 (LSU rDNA), MK671633 (ITS), MT248408 (SSU), MT260392 (*CYTB*), MT260389 (*TEF*), MW036268 (*RPB2*).

Other examined strains: Greenland, Greenland Ice Sheet, 60 km east of Kangerlussuaq, 67° 04′ 43″ N 49° 20′ 29″ W, isolated from dark ice (EXF-12419), isolated from cryoconite (EXF-12886, EXF-12454), July 2017, L. Perini; Norway, Svalbard, Midtre Lovénbreen glacier, isolated from subglacial ice (EXF-12984), SV, Vestre Brøggerbreen glacier, isolated from subglacial ice (EXF-12991), August 2017, L. Perini.

## Supplementary Data

Supplementary material 1
