# Supplementary material for: Greenland and Svalbard glaciers host unknown basidiomycetes: the yeast Camptobasidium arcticum sp. nov. and the dimorphic Psychromyces glacialis gen. and sp. nov
Source: Int J Syst Evol Microbiol. 2021 Jan 27;71(2):004655. doi: 10.1099/ijsem.0.004655 (PMC8346769; doi:10.1099/ijsem.0.004655)

# SUPPLEMENTARY MATERIAL

**Article title: Greenland and Svalbard glaciers host unknown basidiomycetes: the yeast *Camptobasidium arcticum* sp. nov. and the dimorphic *Psychromyces glacialis* gen. and sp. nov.**

**Authors:** Laura Perini[1], Kristina Andrejašič[1], Cene Gostinčar[1,2], Nina Gunde-Cimerman[1], Polona Zalar[1,*]

[1] Department of Biology, Biotechnical Faculty, University of Ljubljana, Večna pot 111, 1000 Ljubljana, Slovenia; [2] Lars Bolund Institute of Regenerative Medicine, BGI-Qingdao, Qingdao 266555, China

*Corresponding author: Polona Zalar, polona.zalar@bf.uni-lj.si

**TABLES:**

Table S1. List of strains considered in the present study, their isolation source, GenBank accession numbers of: LSU rDNA, ITS rDNA, SSU rDNA, cytochrome B (CYTB), translation elongation factor 1 alpha (TEF), and two subunits of the RNA polymerase II (RPB1 and RPB2). http://www.ncbi.nlm.nih.gov.

Table S2. Results on assimilation tests of *Camptobasidium gelus*, *C, arcticum*, and *Psychromyces glacialis*.

**FIGURES:**

Figure S1. Maximum likelihood phylogeny of the concatenated rDNA genes (SSU, LSU, 5.8 S rDNA), estimated by PhyML. aLRT as Chi2 based support was used for calculation of branch supports.

Figure S2. Phylogenetic trees based on alignment of ITS rDNA, estimated by Bayesian inference. The tree includes representatives of (A) *Camptobasidium*., unrooted, and (B) *Psychromyces glacialis* gen. et sp. nov. rooted with *Phenoliferia psychrophenolica*. A total 10 million generations were calculated in each case and the first 25% were discarded before the shown consensus trees were calculated from trees sampled every 100 generations. The numbers given on branches are frequencies at which the individual clades appeared in the sampled trees.

Figure S3. Maximum likelihood tree based on ITS2 sequences obtained in NGS study of glacial environments in Svalbard and the Greenland Ice Sheet, and on ITS sequences of *Camptobasidium gelus*, *Camptobasidium arcticum*, *Camptobasidium* sp. and *Psychromyces glacialis* pure cultures.

Figure S4. Minimum spanning network calculated from the alignment of *Camptobasidium* ITS sequences. The similarity between the strains is shown with the thickness of the lines connecting the circles representing the strains.

Figure S5. Minimum spanning network calculated from the alignment of *Psychromyces* ITS sequences. The similarity between the strains is shown with the thickness of the lines connecting the circles representing the strains.

Figure S6. Morphology of *Psychromyces glacialis* EXF-12419
(a–b): Cultures of *P. glacialis* EXF-12419 on PDA at 15 °C: (a) after 14 days, (b) after 8 months; (c–e) synnemata and hyphae after 14 days in 60% lactic acid; (k) teliospores of EXF-12454 after 2 months; (f, g–j, l) hyphae with clamps stained with calcofluor white after 14 days (f, g, i) and after 2 months (h, j, l).

**Table S1**. List of strains considered in the present study, their isolation source, GenBank accession numbers of: LSU rDNA, ITS rDNA, SSU rDNA, cytochrome B (CYTB), translation elongation factor 1 alpha (TEF), and two subunits of the RNA polymerase II (RPB1 and RPB2). http://www.ncbi.nlm.nih.gov.

| EXF-accession number (*) | Isol. medium | Geographical origin | Isolation source | GenBank Accession numbers | | | | |
|---|---|---|---|---|---|---|---|---|
| | | | | LSU rDNA | ITS rDNA | SSU rDNA | CYTB/TEF | RPB1/ RPB2 |
| ***Camptobasidium gelus*** | | | | | | | | |
| 12745$^{T}$ | MM | GrIS | Cryoconite | MK454802 | MN983240 | MT248410 | -/- | -/MT260387 |
| 12575 | SNA | SV, Midtre Lovénbreen | Subglacial ice | MK670456 | - | - | - | - |
| 12576 | MM | SV, Midtre Lovénbreen | Subglacial ice | MK670454 | MN983222 | MW036178 | -/MW122989 | -/MW036260 |
| 12582 | DRBC | SV, Pedersenbreen | Subglacial ice | MK670478 | MN983223 | - | - | - |
| 12584 | DRBC | SV, Pedersenbreen | Subglacial ice | MK670480 | MN983224 | - | - | - |
| 12594 | DRBC | SV, Vestre Brøggerbreen | Subglacial ice | MK670498 | MN983225 | MW036179 | -/- | MW122991/ MW036261 |
| 12597 | DRBC | SV, Vestre Brøggerbreen | Subglacial ice | MK670502 | MN983226 | - | - | - |
| 12948 | SNA | SV, Midtre Lovénbreen | Subglacial ice | MK670457 | MN983227 | - | - | - |
| 12950 | SNA | SV, Midtre Lovénbreen | Subglacial ice | MK670455 | - | - | - | - |
| 12952 | MM | SV, Midtre Lovénbreen | Subglacial ice | MK670453 | MN983228 | - | - | - |
| 12959 | SNA | SV, Pedersenbreen | Subglacial ice | MK670474 | - | - | - | - |
| 12967 | DRBC | SV, Vestre Brøggerbreen | Subglacial ice | MK670494 | MN983229 | - | - | - |
| 12968 | DRBC | SV, Vestre Brøggerbreen | Subglacial ice | MK670495 | MN983230 | - | - | - |
| 12969 | DRBC | SV, Vestre Brøggerbreen | Subglacial ice | MK670492 | - | - | - | - |
| (12970) | MM | SV, Vestre Brøggerbreen | Subglacial ice | MK670500 | MN983231 | - | - | - |
| 13102 | MM | SV | Glacial meltwater | MK670451 | MN983232 | MW036180 | -/- | MW122993/ MW036262 |
| 13103 | MM | SV, Pedersenbreen | Subglacial ice | MK670469 | MN983233 | MW036177 | -/- | MW122992/ MW036267 |
| 13104 | SNA | SV, Pedersenbreen | Subglacial ice | MK670473 | - | - | - | - |

| | | | | | | | | |
|---|---|---|---|---|---|---|---|---|
| 13113 | SNA | SV, Vestre Brøggerbreen | Subglacial ice | MK670503 | MN983234 | - | - | - |
| (12394) | R2A | GrIS | Supraglacial water | MK454824 | - | - | - | - |
| 12401 | R2A | GrIS | Dark ice | MK454807 | MN983235 | - | - | - |
| 12406 | R2A | GrIS | Dark ice | MK454810 | MN983236 | - | - | - |
| 12527 | R2A | GrIS | Clear ice | MK454796 | - | - | - | - |
| 12693 | R2A | GrIS | Supraglacial water | MK454821 | - | - | - | - |
| (12694) | R2A | GrIS | Supraglacial water | MK454817 | MN983237 | - | - | - |
| (12698) | R2A | GrIS | Supraglacial water | MK454823 | - | - | - | - |
| 12712 | SNA | GrIS | Clear ice | MK454797 | MN983238 | - | - | - |
| 12714 | SNA | GrIS | Clear ice | MK454799 | - | - | - | - |
| (12716) | SNA | GrIS | Clear ice | MK454801 | - | - | - | - |
| 12717 | R2A | GrIS | Clear ice | MK454794 | MN983239 | - | - | - |
| 15272 | SNA | GrIS | Dark ice | MK454811 | - | - | - | - |
| 15273 | R2A | GrIS | Clear ice | MK454795 | - | - | - | - |
| ***Camptobasidium arcticum*** | | | | | | | | |
| 12713[T] | SNA | GrIS | Clear ice | MK454798 | MN983248 | MT304813 | MT260394/ MT260390 | -/ MT260386 |
| (12392) | R2A | GrIS | Supraglacial water | MK454819 | MN983241 | - | - | - |
| (12405) | R2A | GrIS | Dark ice | MK454808 | - | - | - | - |
| 12516 | R2A | GrIS | Dark ice | MK454809 | MW019434 | - | - | - |
| 12519 | MM | GrIS | Supraglacial water | MK454812 | MW019433 | - | - | - |
| 12522 | R2A | GrIS | Supraglacial water | MK454816 | MW019432 | MW036182 | MW036274/- | -/ MW036263 |
| 12524 | R2A | GrIS | Supraglacial water | MK454818 | MN983242 | MW036181 | MW036272/ MW122987 | MW122995/ MW036264 |
| 12686 | SNA | GrIS | Supraglacial water | MK454827 | MN983243 | - | - | - |
| 12687 | MM | GrIS | Supraglacial water | MK454813 | MN983244 | - | - | - |
| (12688) | MM | GrIS | Supraglacial water | MK454814 | - | - | - | - |
| 12689 | MM | GrIS | Supraglacial water | MK454815 | MN983245 | - | - | - |
| (12692) | R2A | GrIS | Supraglacial water | MK454820 | MN983246 | - | - | - |
| 12696 | R2A | GrIS | Supraglacial water | MK454825 | - | - | - | - |
| (12697) | R2A | GrIS | Supraglacial water | MK454822 | MN983247 | - | - | - |
| 12711 | DRBC | GrIS | Dark ice | MK454805 | MW019431 | MW036183 | MW036273/ MW122988 | MW122994/ MW036265 |

| | | | | | | | | |
|---|---|---|---|---|---|---|---|---|
| 12715 | SNA | GrIS | Clear ice | MK454800 | MW019430 | - | - | - |
| 12728 | DRBC | GrIS | Dark ice | MK454804 | MN983249 | - | -/ MW122986 | -/ MW036266 |
| (12868) | SNA | GrIS | Cryoconite | MK454803 | MN983250 | - | - | - |
| 12872 | R2A | GrIS | Supraglacial water | MK454826 | MN983251 | - | - | - |
| 13086 | R2A | GrIS | Clear ice | MK454828 | MN983252 | - | - | - |
| (15271) | R2A | GrIS | Dark ice | MK454806 | MN983253 | - | - | - |
| ***Camptobasidium* sp.** | | | | | | | | |
| (12685) | SNA | GrIS | Supraglacial water | MK454829 | MN983221 | MT248411 | - | - |
| ***Psychromyces glacialis*** | | | | | | | | |
| 13111[T] =CBS 16467 | DRBC | SV, Vestre Brøggerbreen | Suglacial ice | MT301949 | MK671633 | MT248408 | MT260392/ MT260389 | -/ MW036268 |
| 12419 | MM | GrIS | Dark ice | MK454861 | MK460401 | MT248407 | MT260391/- | -/ MW036270 |
| 12886 | DG18 | GrIS | Cryoconite | MT301950 | MK460394 | MT248409 | MT260393/ MT260388 | -/ MW036269 |
| 12398 | DRBC | GrIS | Clear ice | MK454857 | MK460391 | - | - | - |
| 12420 | MM | GrIS | Dark ice | - | MK460402 | - | - | - |
| 12452 | MM | GrIS | Cryoconite | MK454858 | MK460396 | - | - | - |
| 12454 | MM | GrIS | Cryoconite | MK454859 | MK460397 | - | - | - |
| 12545 | MM | GrIS | Cryoconite | - | MK460395 | - | - | - |
| 12604 | DRBC | GrIS | Dark ice | MK454862 | MK460403 | - | - | - |
| 12623 | MM | SV, Midtre Lovénbreen | Suglacial ice | - | MK671639 | - | - | - |
| 12626 | DRBC | SV, Midtre Lovénbreen | Suglacial ice | - | MK671638 | - | - | - |
| 12627 | SNA | SV, Midtre Lovénbreen | Suglacial ice | - | MK671636 | - | - | - |
| 12647 | DRBC | SV, Vestre Brøggerbreen | Suglacial ice | - | MK671634 | - | - | - |
| 12708 | DRBC | GrIS | Dark ice | MK454860 | MK460399 | - | - | - |
| 12718 | DRBC | GrIS | Clear ice | MW026145 | MK460392 | - | - | - |
| 12890 | DG18 | GrIS | Cryoconite | - | MK460398 | - | - | - |
| 12984 | DRBC | SV, Midtre Lovénbreen | Suglacial ice | MW026144 | MK671637 | - | - | - |
| 12991 | DRBC | SV, Vestre Brøggerbreen | Suglacial ice | MW026141 | MK671635 | MW036176 | MW036271/ MW122990 | - |
| 13154 | DRBC | GrIS | Dark ice | MW026143 | MK460400 | - | - | - |
| 13156 | SNA | GrIS | Clear ice | MW026142 | MK460393 | - | - | - |

Legend: GrIS: Greenland Ice Sheet, SV: Svalbard; (*) the accession numbers of strains that didn't survive freezing period of 3 years at -80 °C are written in the brackets.

**<u>Table S2.</u>** Results on assimilation tests of *Camptobasidium gelus, C. arcticum*, and *Psychromyces glacialis*.

| | C-SOURCES | ***Camptobasidium gelus*** | ***Camptobasidium arcticum*** | ***Psychromyces glacialis* (EXF-13111-yeast)** | ***Psychromyces glacialis* (filamentous)** |
|---|---|---|---|---|---|
| Hexose-based carbon sources | **a-D-glucose** | + | + | + | +,D; YT:- |
| | inulin | V(-,+) | - | - | - |
| | **sucrose** | + | + | + | + |
| | **D-raffinose** | + | D | + | +, YT:-, D |
| | **D-melibiose** | - | V(+,-), YT:- | - | - |
| | **D-galactose** | V (-,W, D) | - | D | - |
| | **Lactose** | V(-,W) | V(-,+,W) | - | V(-, WD) |
| | **D-trehalose** | V(-,+,W) | +,W; YT:- | + | D |
| | **maltose** | W,+; YT:- | + | D | +, YT: W, D |
| | **D-melezitose** | - | + | + | + |
| | a-methyl D-glucoside | - | - | - | - |
| | **Starch** | - | - | - | - |
| | **cellobiose** | - | + | W; YT:- | W,+ |
| | **salicin** | W,+ | V(-,W) | W,D; YT:- | V(-, WD) |
| | **L-sorbose** | V(-,W,D) | - | + | - |
| | L-rhamnose | - | - | - | - |
| | gentiobiose | - | + | - | V(+,-) |
| | maltotriose | - | + | D | V(+,-) |
| | D-psicose | - | - | - | - |
| | palatinose | - | V(+,-,D) | - | - |
| | stachyose | + | D | + | V(-,D) |
| | b-methyl D-glucoside | - | + | - | V(-,D) |
| | turanose | - | - | - | - |
| | dextrin | - | +,D | D | D |
| | arbutin | - | - | - | - |
| | amygdalin | - | - | - | - |
| pentose | **D-xylose** | W,D | V(-, W, D) | W,D | - |
| | **L-arabinose** | - | - | - | V(-,W) |
| | **D-arabinose** | - | - | - | - |
| | **D-ribose** | D, W, + | - | - | - |
| polyols | **glycerol** | V(-, W,+), YT: - | - | - | - |
| | i-erythritol | - | - | - | - |
| | xylitol | - | - | - | - |
| | D-mannitol | - | - | - | - |
| | D-sorbitol | - | - | W,D | - |
| | maltitol | - | V(+,-,W,D) | - | - |
| | adonitol (ribitol) | - | - | - | - |
| | D-arabitol | - | - | - | - |
| acids | methyl succinate | - | - | - | - |
| | fumaric acid | - | - | - | V(+,-) |
| | L-malic acid | - | - | - | - |

| | | | | | |
|---|---|---|---|---|---|
| | bromo succinic acid | - | - | - | - |
| | L-glutamic acid | - | - | - | V(-,W) |
| | g-amino butyric acid | V(D,-) | W | D | - |
| | D-gluconic acid | - | - | - | - |
| | a-keto-glutaric acid | - | - | - | - |
| | 2-keto-D-gluconic acid | - | - | - | - |
| glucose based amines | **D-glucosamine** | **-** | **-** | **W,D** | **-** |
| | N-acetyl-D-glucosamine | - | - | - | - |
| D-xylose with additional sources | methyl succinate + D-xylose | - | - | - | - |
| | N-acetyl-L-glutamic acid + D-xylose | - | V(+,-,W,D) | D | - |
| | quinic acid + D-xylose | - | V(+,-,W,D) | D | - |
| | D-glucuronic acid + D-xylose | D | V(+,-,D) | + | - |
| | dextrin + D-xylose | - | +, D | + | V(-, D) |
| | a-D-lactose + D-xylose | - | V(+,-,D) | - | V(-, D) |
| | D-melibiose + D-xylose | - | V(+,-,W,D) | D | - |
| | D-galactose + D-xylose | V(-, W,D) | V(+,-,W,D) | + | - |
| | m-inositol + D-xylose | - | V(+,-,W,D) | - | - |
| | 1,2-propanediol + D-xylose | - | V(+,-,D) | W,D | - |
| | acetoin + D-xylose | - | - | W,D | - |
| | **N-SOURCES** | | | | |
| | **nitrate** | **+** | **V(+,-,W)** | **+** | **+** |
| | **nitrite** | **V(-,W)** | **-** | **+** | **+/WD** |
| | **L-lysine** | **+** | **V(W,-)** | **W** | **V(W,-)** |
| | **cadaverine** | **+** | **+,WD** | **+** | **+** |
| | **OTHER TESTS** | | | | |
| | tween 80 | V(-, W,+) | - | + | + |
| | **10% NaCl** | **-** | **-** | **-** | **-** |
| | **15% NaCl** | **-** | **-** | **-** | **-** |
| | **50% glc** | **-** | **-** | **-** | **-** |

LEGEND:

**black, bold: classic assimilation, Biolog tests in agreement**

**red, bold: classic assimilation, BIOLOG YT tests NOT in agreement**

normal, black: Biolog YT tests only

**blue, bold: classic assimilation tests only**

+: assimilation, -: no assimilation, w: weak assimilation, v: variable results, d: delayed assimilation, YT - BIOLOG YT plate results

**Figure S1**. Maximum likelihood phylogeny of the concatenated rDNA genes (SSU, LSU, 5.8 S rDNA), estimated by PhyML. aLRT as Chi2 based support was used for calculation of branch supports.

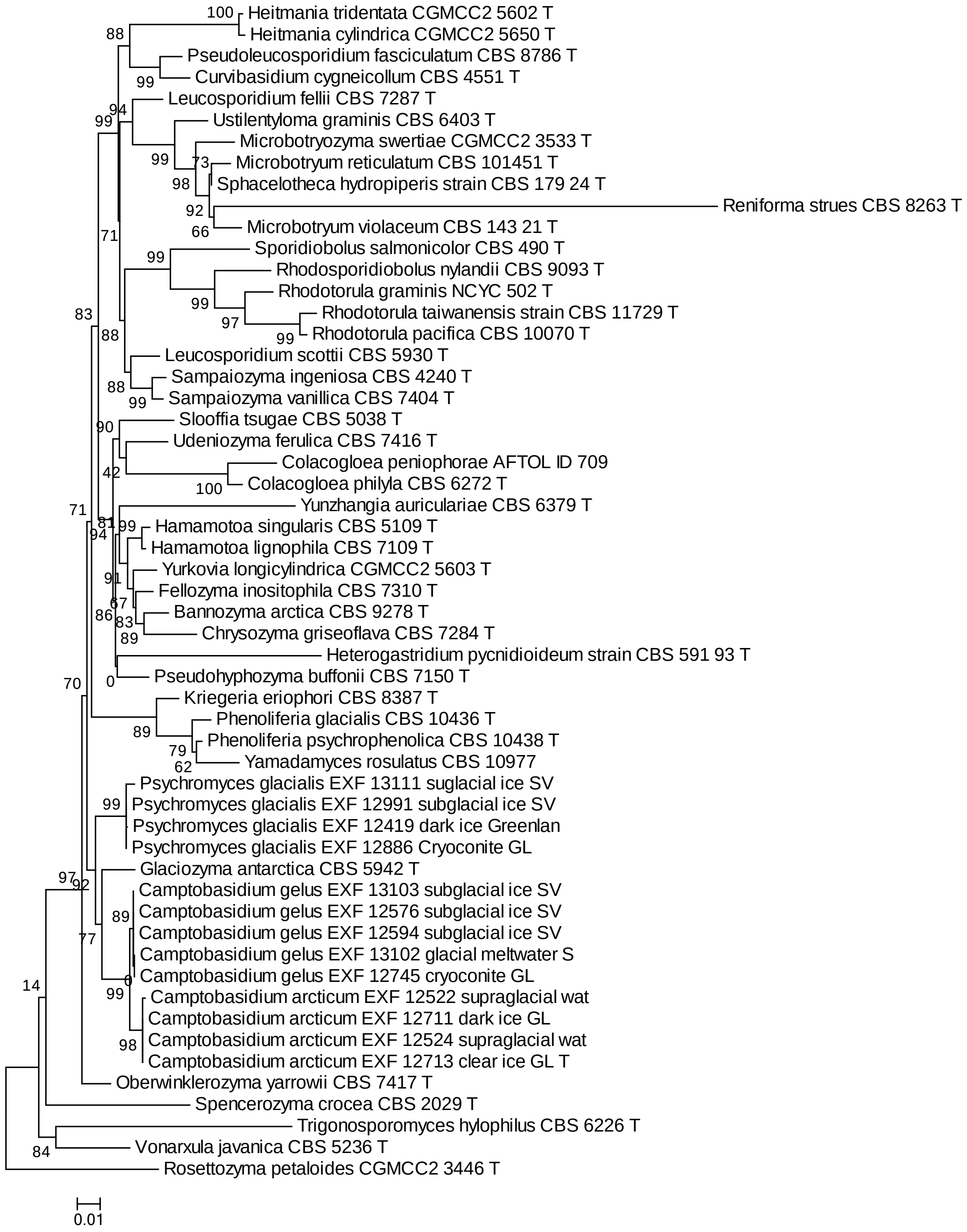

**<u>Figure S2.</u>** Phylogenetic trees based on alignment of ITS rDNA, estimated by Bayesian inference. The tree includes representatives of (A) *Camptobasidium*. unrooted, and (B) *Psychromyces glacialis* gen. et sp. nov. rooted with *Phenoliferia psychrophenolica*. A total 10 million generations were calculated in each case and the first 25% were discarded before the shown consensus trees were calculated from trees sampled every 100 generations. The numbers given on branches are frequencies at which the individual clades appeared in the sampled trees.

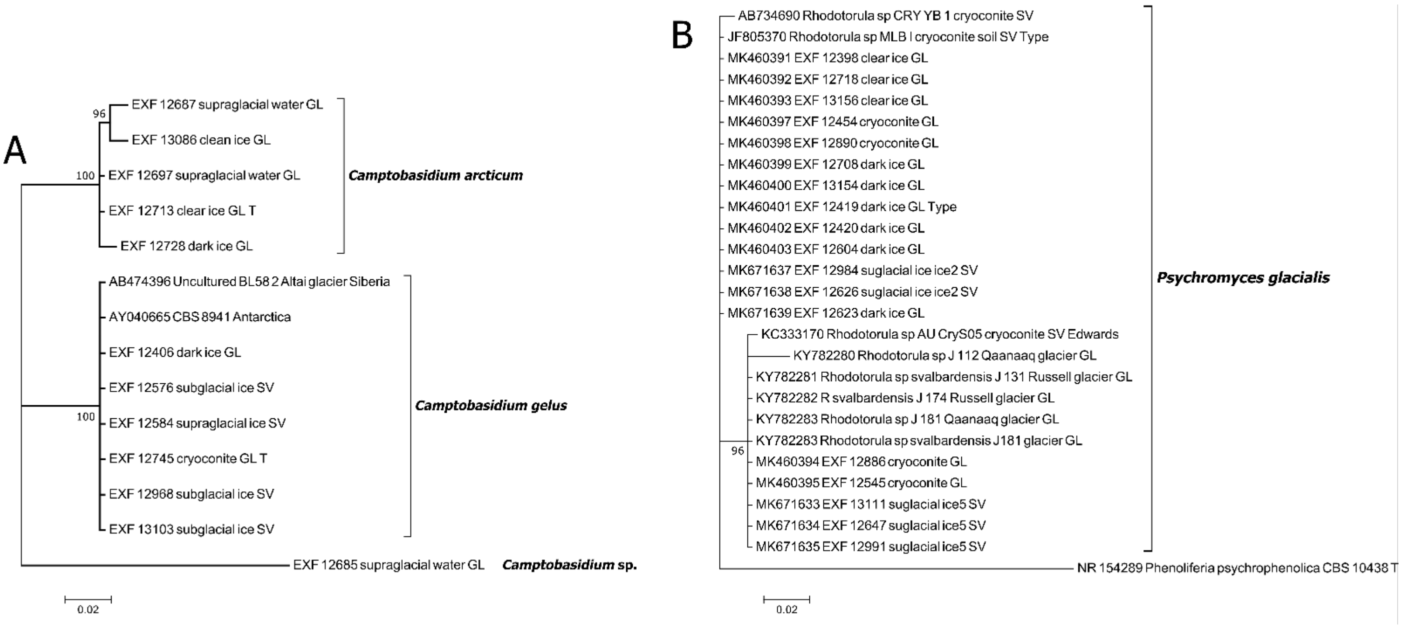

**Figure S3**. Maximum likelihood tree based on ITS2 sequences obtained in NGS study of glacial environments in Svalbard and the Greenland Ice Sheet, and on ITS sequences of *Camptobasidium gelus, Camptobasidium arcticum, Camptobasidium* sp. and *Psychromyces glacialis* pure cultures.

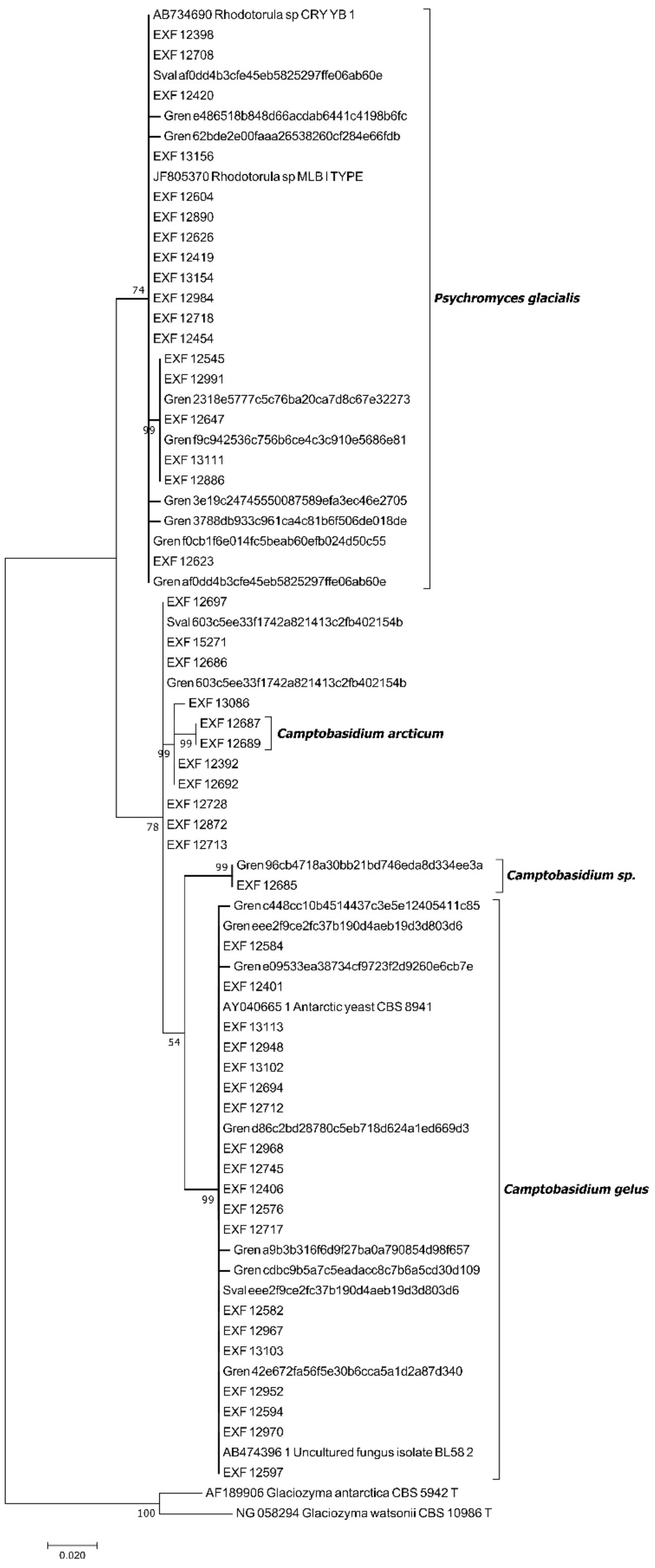

**Figure S4.** Minimum spanning network calculated from the alignment of *Camptobasidium* ITS sequences. The similarity between the strains is shown with the thickness of the lines connecting the circles representing the strains.

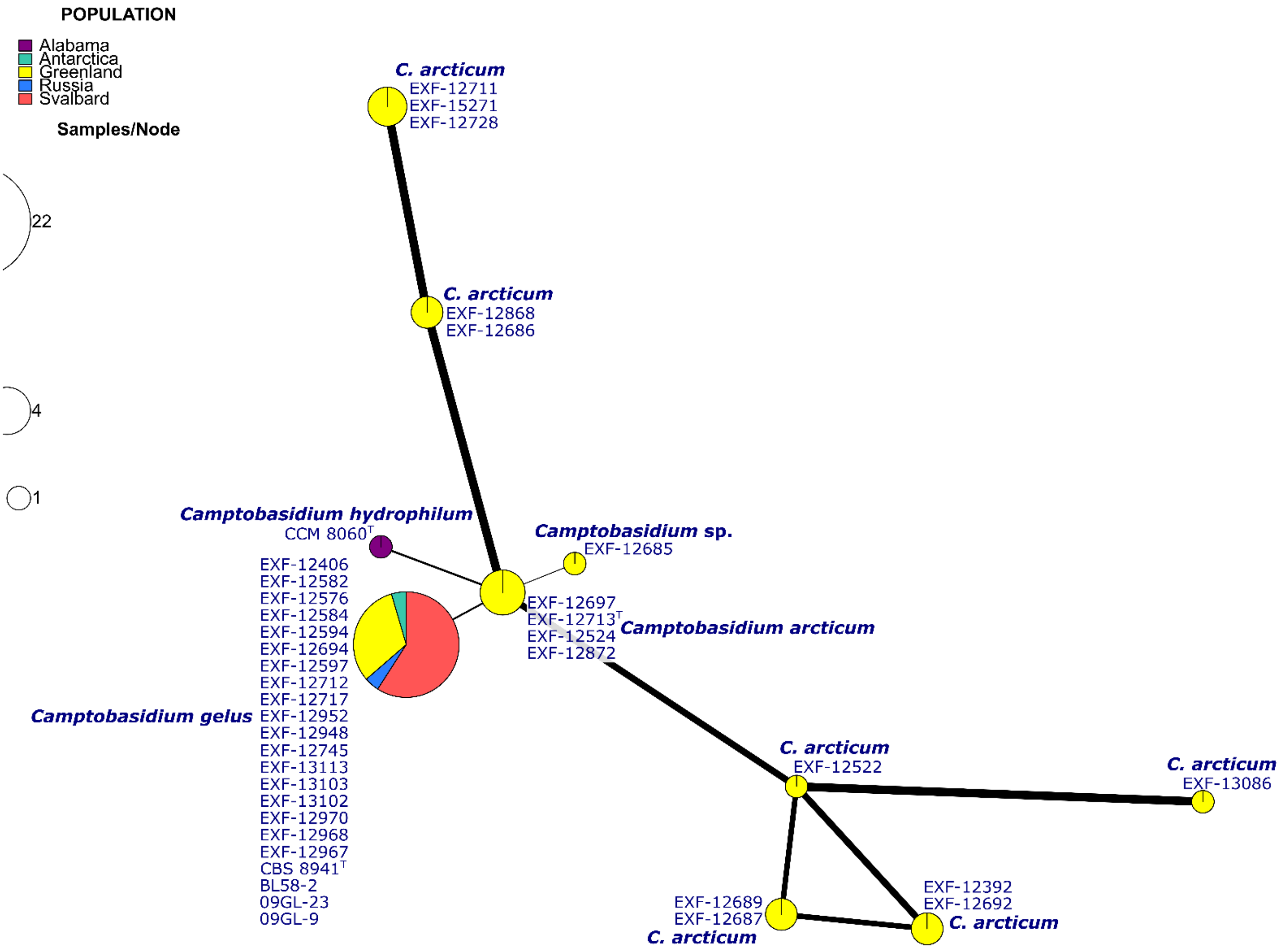

**<u>Figure S5</u>**. Minimum spanning network calculated from the alignment of *Psychromyces* ITS sequences. The similarity between the strains is shown with the thickness of the lines connecting the circles representing the strains.

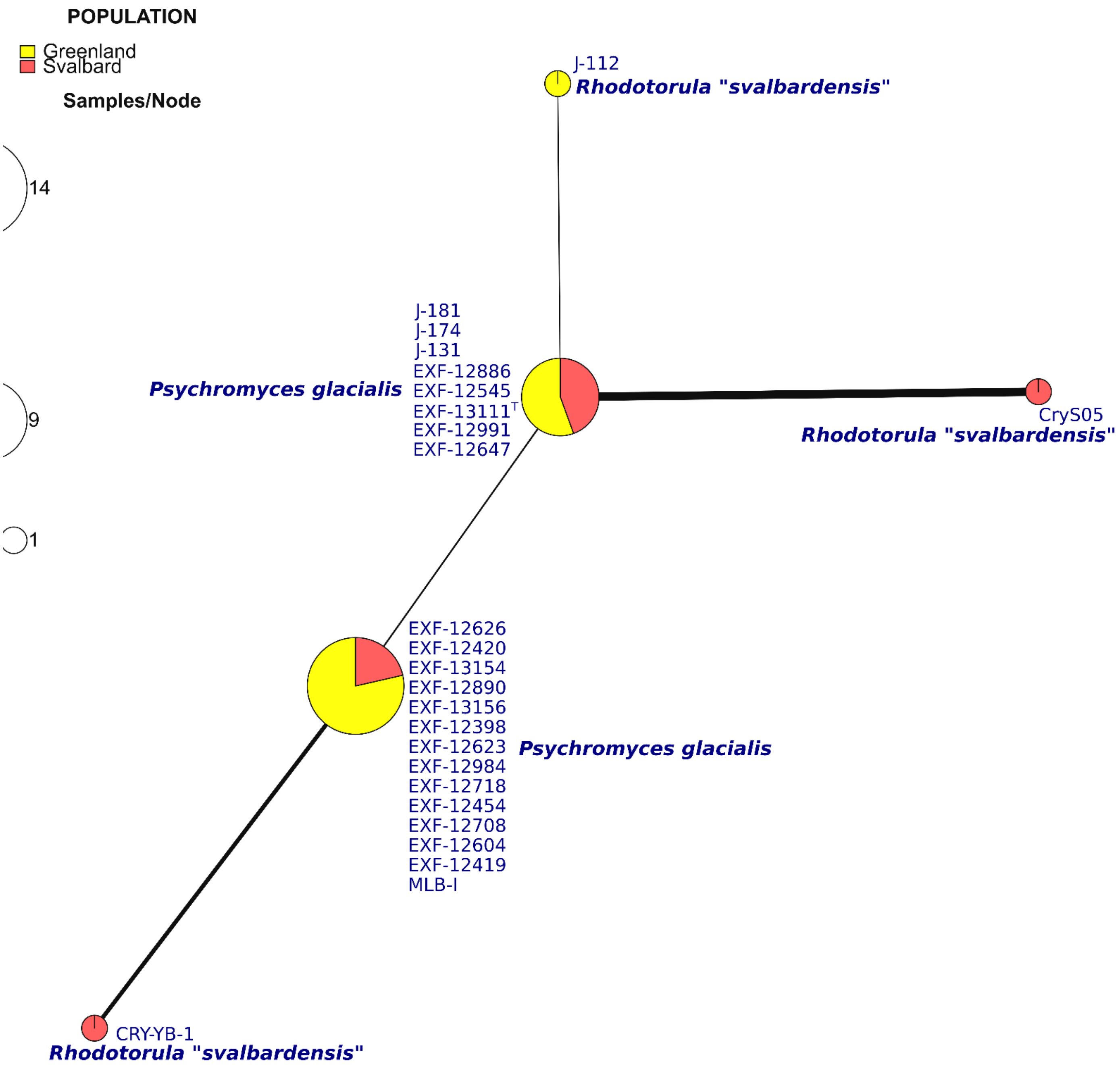

**<u>Figure S6.</u>** Morphology of *Psychromyces glacialis* EXF-12419
X5 (a–b): Cultures of *Psychromyces glacialis* EXF-12419 on PDA at 15 °C: (a) after 14 days, (b) after 2 months; (c–e) synnemata and hyphae after 14 days in 60% lactic acid; (k) teliospores of EXF-12454 after 2 months; (f, g–j, l) hyphae with clamps stained with calcofluor white after 14 days (f, g, i) and after 2 months (h, j, l).

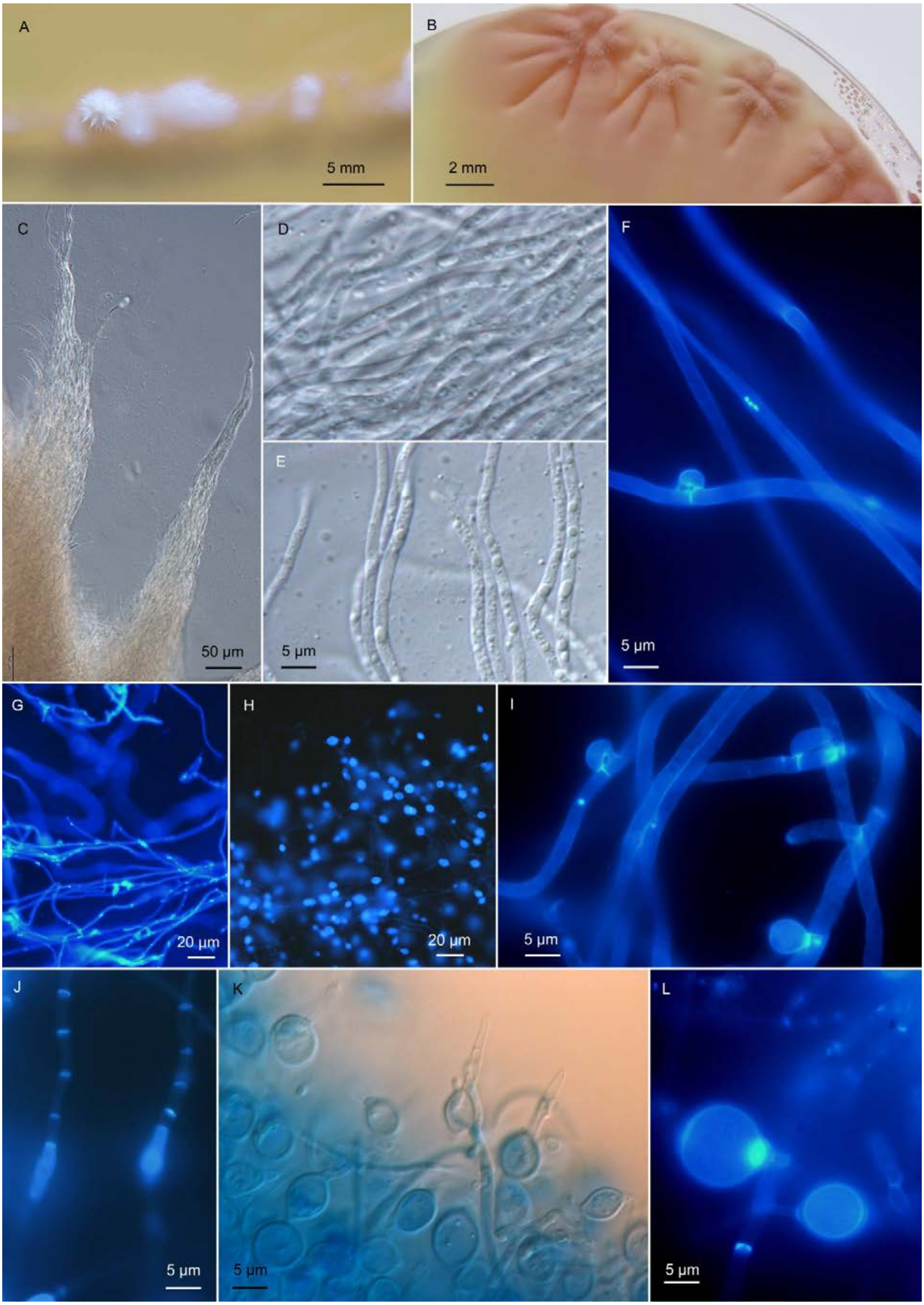

Supplement: Supplementary material 1 [file ijsem-71-655-s001.pdf]
